# Characterization of a Novel MMS-Sensitive Allele of *Schizosaccharomyces pombe mcm4^+^*

**DOI:** 10.1534/g3.116.033571

**Published:** 2016-07-29

**Authors:** Nimna S. Ranatunga, Susan L. Forsburg

**Affiliations:** Program in Molecular and Computational Biology, University of Southern California, Los Angeles, California 90089

**Keywords:** fission yeast, DNA replication, MCM complex, MMS, checkpoint, fork protection complex

## Abstract

The minichromosome maintenance (MCM) complex is the conserved helicase motor of the eukaryotic replication fork. Mutations in the Mcm4 subunit are associated with replication stress and double strand breaks in multiple systems. In this work, we characterize a new temperature-sensitive allele of *Schizosaccharomyces pombe mcm4^+^*. Uniquely among known *mcm4* alleles, this mutation causes sensitivity to the alkylation damaging agent methyl methanesulfonate (MMS). Even in the absence of treatment or temperature shift, *mcm4-c106* cells show increased repair foci of RPA and Rad52, and require the damage checkpoint for viability, indicating genome stress. The *mcm4-c106* mutant is synthetically lethal with mutations disrupting fork protection complex (FPC) proteins Swi1 and Swi3. Surprisingly, we found that the deletion of *rif1^+^* suppressed the MMS-sensitive phenotype without affecting temperature sensitivity. Together, these data suggest that *mcm4-c106* destabilizes replisome structure.

The MCM helicase comprises six related proteins (Mcm2–7) that form a highly conserved heterohexameric ring functioning as the primary unwinding activity in the eukaryotic replisome (reviewed in Abid Ali and Costa 2015; O’Donnell and Li 2016). The loading of the MCM complex specifies potential replication origins. Replication initiation occurs with assembly of active replicative helicase known as CMG (Cdc45-Mcm-GINS). The helicase travels with other replisome components (Gambus *et al.* 2009). Recent studies examining the structure of the active CMG complex have provided insight into its mechanism. Cdc45 and GINS are activating agents (Moyer *et al.* 2006; Ilves *et al.* 2010) that bind at the Mcm2-Mcm5 “gate” where the MCM ring opens and closes around DNA (Costa *et al.* 2011, 2014; Sun *et al.* 2015). CMG makes direct contacts at the leading C-terminal side of the MCM ring with DNA polymerase ε, which is the processive leading strand polymerase (Langston *et al.* 2014; Sun *et al.* 2015). The DNA polymerase α/primase complex that initiates lagging strand synthesis is coupled to CMG via a trimeric protein called Mcl1 (Sc Ctf4, Hs AND-1) (Simon *et al.* 2014). Another conserved protein, Mrc1 (Hs Clapsin), is thought to help maintain the coupling with DNA polymerase Epsilon (Lou *et al.* 2008). The Swi1-Swi3 complex (Hs Timeless-Tipin, Sc Tof1-Csm3), called the Fork Protection Complex (FPC), also acts with Mrc1 and travels with the replisome (reviewed in Leman and Noguchi 2012). The FPC is not essential for viability in the yeasts, but in its absence, cells show uncoupling of the replisome and increased ssDNA formation, disruption in activation of the replication checkpoint, sensitivity to DNA damaging agents, and defects in cohesin (reviewed in Leman and Noguchi 2012).

The Mcm4 subunit resides on the opposite side of the MCM ring from the Mcm2–5 gate that binds Cdc45 and GINS, near the proposed lagging strand template (reviewed in O’Donnell and Li 2016). Interestingly, numerous mutations in this subunit have been linked to genome instability in mammalian systems. The point mutation F345I (*chaos3*), located downstream of the Zn-finger motif of MCM4 in mouse, is associated with mammary carcinoma (Shima *et al.* 2007). The *mcm4-D573H* mutation is associated with T cell lymphoblastic leukemia/lymphoma in a mouse model (Bagley *et al.* 2012), and *mcm4-G364R* in humans is associated with skin cancer (Ishimi and Irie 2015). All these mutations are related with increased double strand breaks, and in some cases formation of micronuclei. N-terminally truncated Mcm4 (∆1–50) is linked to glucocorticoid deficiency and defective DNA repair in humans (Hughes *et al.* 2012; Gineau *et al.* 2012). Although the primary sequence of the Mcm4 N-terminus is neither conserved nor essential, this domain appears to be a common substrate for the DDK kinase required to initiate replication (Masai *et al.* 2006; Sheu and Stillman 2006). In budding yeast, deletion of the N-terminus bypasses a requirement for DDK, suggesting that DDK overcomes an inhibitory function (Sheu and Stillman 2010). The N-terminus is also important for regulating fork progression when cells have depleted nucleotide pools during hydroxyurea (HU) treatment (Devault *et al.* 2008; Sheu *et al.* 2014). C-terminal truncations of Mcm4 also cause HU sensitivity, and fail to restrain single-stranded DNA accumulation (Nitani *et al.* 2008).

The fission yeast *mcm4^+^* (*cdc21^+^)* gene was originally identified in a screen for temperature-sensitive *cdc* mutants that arrest as elongated cells with undivided nuclei at the restrictive temperature (Nasmyth and Nurse 1981; Coxon *et al.* 1992). These *mcm4-M68* cells accumulate approximately 2C DNA content, and show evidence of DNA damage including DNA double strand breaks and generating a robust checkpoint-dependent arrest (Nasmyth and Nurse 1981; Coxon *et al.* 1992; Liang *et al.* 1999; Bailis *et al.* 2008; Sabatinos *et al.* 2015). Viability is low upon return to permissive temperature, suggesting that this damage is irreversible (Liang *et al.* 1999; Bailis *et al.* 2008). The 2C DNA content observed in *mcm4-M68* suggests that cells are competent for replication initiation and the bulk of DNA replication at the restrictive temperature. A second temperature-sensitive allele was constructed by fusing a *degron* cassette to *mcm4-M68* (Lindner *et al.* 2002). This enhances protein turnover leading to a rapid inactivation at restrictive temperature, and cells arrest with a 1C DNA content (Lindner *et al.* 2002; Bailis *et al.* 2008; Sabatinos *et al.* 2015). However, despite evidence of DNA damage including large RPA and Rad52-containing “megafoci,” *mcm4-dg* cells continue to divide, indicating that they have evaded the damage checkpoint (Sabatinos *et al.* 2015). Survivors show dramatic evidence for chromosome mis-segregation, abnormal nuclear division, and chromosome rearrangement (Sabatinos *et al.* 2015).

A large C-terminal truncation mutant, *mcm4-c106*, is both temperature-sensitive and HU-sensitive (Nitani *et al.* 2008). In this study, we show that, unlike other mutant alleles of *mcm4*, *mcm4-c106* is also sensitive to the alkylating agent MMS. Moreover, the phenotype of *mcm4-c106* at the restrictive temperature with high viability is distinct from that of the other temperature-sensitive alleles. Genetic interactions and synthetic lethality with components of the fork protection complex suggest that the *mcm4-c106* truncation mutation leads to specific defects in maintaining replisome structure, causing genome instability.

## Materials and Methods

### Cell growth and cultures

Fission yeast strains are listed in Table 1. All strains were maintained according to standard protocols (Sabatinos and Forsburg 2010). Strains were grown in YES or Edinburgh minimal medium (EMM) with ammonium chloride as the nitrogen source, supplemented with the required nutrients at 25° unless otherwise stated. For all experiments, cultures were grown in 5 ml of liquid media from a single colony at 25° overnight and released to fresh media and grown at 25° to midlog phase. Serial dilutions and plating assays were performed in cultures grown in YES, while the imaging experiments were performed in cultures grown in EMM.

**Table 1:** Strains used in this study

Strain	Genotype	Source
FY 7	*h- 972*	Our stock
FY 527	*h-his3-D1 ade6-M216 ura4-D18 leu1-32*	Our stock
FY 528	*h+ his3-D1 ade6-M210 ura4-D18 leu1-32*	Our stock
FY 261	*h+ can1-1 leu1-32 ade6-M216 ura4-D18*	Our stock
FY 784	*h+ cdc21-M68 ura4-D18 leu1-32 ade6-M210 can1-1 (mcm4)*	Our stock
FY 4241	*h- cdc21-c106:kan*	Takuro Nakagawa
FY 4311	*h- cdc21-c106*::*kan ura4-D18 his3-D1 ade6- M210*	Our stock
FY 4310	*h- cdc21-c84*::*kan Ura4-D18 his3-D1*	Our stock
FY 5942	*h- cdc21-c106*::*HphMx ura4-D18 his3-D1 ade6- M210*	This work
FY 3395	*h- mcm4(cdc21-M68)-ts-dg*::*ura4+ ura4-D18*	Our stock
FY 6126	*h+ cdc21-c106*::*kan ura4-D18 his 3D-1 leu1-32 ade6-M210*	Our stock
FY 6038	*h- pcn1-K164R*::*ura4 cdc21-c106*::*kan ura4-D18 ade6-M210*	Our stock
FY 6039	*h+kpa1*Δ::*bleMX6 cdc21-c106*::*kan his4- 239/his3-D1 ade6-M26 ?(kpa1)*	Our stock
FY 6040	*h+*Δ*rhp18*::*ura4 cdc21-c106*::*kan ura4-D18 leu1- 32 ade6-704*	This work
FY 6041	*h*- Δ*reb1*::*kanMX cdc21-c106*::*HphMx his 3-D1 ura4-D18 ade6-M210*	This work
FY 6042	*h+*Δ*reb1*::*kanMX cdc21-c106*::*HphMx ura4-D18 leu1-32 ade6-M216*	This work
FY 6043	*h- cdc21-c106*::*kan cyc17*::*ura4 his3 D-1 ade6- M216 ura4 cyc17 = allelic to cig2*	This work
FY 6044	*h+ cdc21-c106*::*kan cyc17*::*ura4 his3 D-1 ade6- M216 leui1-32 ura4*	This work
FY 6052	*h+* Δ*rev1*::*ura4+ cdc21-c106*::*kan ura4-D18 his3-D1 ade6-M216/210?ura4-D18*	This work
FY 6053	*h*- Δ*rev1*::*ura4+ cdc21-c106*::*kan ura4-D18 his3? ade6-M?ura4-D18*	This work
FY 6054	*h- eso1-eta*Δ::*kanMX6 cdc21-c106*::*HphMx ura4- D18 his3-D1 ade6-M210*	This work
FY 6055	*h- eso1-eta*Δ::*kanMX6 cdc21-c106*::*HphMx ura4- D18 his3-D1 ade6-M210 Leu1-32*	This work
FY 6077	*h+* Δ*rad8*::*hphMX cdc21-c106*::*kan ura4-D18 his3-D1 ade6-M216 leu1-32*	This work
FY 6078	*h*- Δ*brc1*::*ura4+ cdc21-c106*::*kan ura4-D18 ade6-M210*	This work
FY 6079	*h*-Δ*brc1*::*ura4+ cdc21-c106*::*kan ura4-D18 his 3- D1 ade6-M210*	This work
FY 6080	*h+* Δ*Ubc13*::*ura4+ cdc21-c106*::*kan his3D18 ura4-D18 ade6-m210*	This work
FY 6123	*h- cdc21-c106*::*kan rev3*::*hphMX6 ura4-D18 his3-D1 ade6-M210*	This work
FY 6146	*h+srs2*::*kan cdc21-c106*::*HphMx ade6-M210 leu1-32 ura4-D18 his3-D1*	This work
FY 6147	*h-srs2*::*kan cdc21-c106*::*HphMx ade6-M210 ura4- D18 his3-D1*	This work
FY6238	*h+ cdc21-c106*::*HphMx ura4-D18 his3-D1 leu1- 32 ade6-M210*	This work
FY 6248	*h+*Δ *mms2*::*leu2 cdc21-c106*::*kan ura4-D18 leu1- 32 his4-239 ade6-M26*	This work
FY 6266	*h-cdc21-c106*::*kan rad11-Cerulean*::*hphMX rad22-YFP-natMX ura4-D18 leu1-32 ade6-M210*	This work
FY 6281	*h-cdc21-c106*::*kan chk1HA(ep) ade6-M216 ura4- D18 leu1-32 his 3D-1*	This work
FY 6308	*h+ cdc21-c106*::*kan* Δ*cds1*::*ura4+ ura4-D18 leu1- 32 his3-D1 ade6-M210*	This work
FY 6309	*h- cdc21-c106*::*kan* Δ*cds1*::*ura4+ ura4-D18 his3- D1 ade6-M210*	This work
FY 6751	*h+ cdc21-c106*::*kan mad2D*::*ura4+ ade6-M210 leu1-32 ura4-D18*	This work
FY 6777	*h- cdc21-c106*::*HphMx* Δ*rif1:kanMX6-Bioneer ura4-D18 ade6-M210 his3-D1*	This work
FY 6778	*h+ cdc21-c106*::*HphMx* Δ*rif1:kanMX6-Bioneer ura4-D18 ade6-M210 his3-D1*	This work
FY 6779	*h- cdc21-c106*::*kan exo1*::*ura4 ura4-D18 ura4- D18 ade6-M210*	This work
FY 6780	*h+ cdc21-c106*::*kan exo1*::*ura4 ura4-D18 ura4- D18 ade6-M210*	This work
FY 6961	*h- swi6*::*ura4+ cdc21-c106*::Kan leu1-32 ura4- (DS/E or D18?) ade6-M210 *can1-1*	This work
FY 7045	*h+ cdc21-c106*::*kan fml1*::*natMX4 ura4-D18 his 3D-1 leu1-32 ade6-M210/216?*	This work
FY 7047	*cdc21-c106*::*kan fml1*::*natMX4 ura4-D18 his 3D- 1 leu1-32 ade6-M210/216*	This work
FY 7048	*h- chp1*::*kanMX6-Bioneer cdc21-c106*::*HphMx his3-D1 leu1-32 ura4-D18 ade6-M216/210?*	This work
FY 7165	*h*- Δ*mus81*::*KanMX cdc21-c106*::*HphMx ura4- D18 his3-D1 ade6-M210*	This work
FY 7166	*h+* Δ*mus81*::*KanMX cdc21-c106*::*HphMx ura4- D18 his3-D1 ade6-M210*	This work
FY 7461	*h- mcl1-11 cdc21-c106*::*kan ade6-704 ura4-294 leu1-32 his3D-1*	This work
FY 7462	*h+mcl1-11 cdc21-c106*::*kan ade6-704 ura4-294 leu1-32 his3D-1*	This work
FY7611	*h+ rhp51*::*ura4+ cdc21-c106*::*kan ade6-704/ade 6-M210 leu1-32 ura4-D18*	This work
FY 7802	*h+* Δ*chl1*::*kanMX6-Bioneer cdc21-c106*::*HphMx his3-D1 leu1-32 ura4-D18 ade6-M210*	This work
FY 7922	*h+ arg3+*::*ccr1N-mCherry((D817 aa1-275)*::*his5+ cdc21-c106*::*kan rad11-Cerulean*::*hphMX rad22- YFP-natMX ura4-D18 his5D leu1-32 ade6- M210*	This work
FY 7923	*h*- Δ*rif1*::*ura4+* Δ*swi1*::*KanMX ura4-D18 leu1-32 ade6-M210 his3-D1*	This work
FY 7924	*h+* Δ*rif1*::*ura4+* Δ*swi1*::*KanMX ura4-D18 leu1-32 ade6-M210 his3-D1*	This work
FY 7925	*h*-Δ*rif1*::*ura4+* Δ*swi3*::*KanMX ura4-D18 leu1-32 ade6-M210 his3-D1*	This work
FY 7926	*h+*Δ*rif1*::*ura4+* Δ*swi3*::*KanMX ura4-D18 leu1-32 ade6-M210 his3-D1*	This work
FY 3664	*h+ mcm4-”chaos” ura4-D18 leu1-32 ade6-M210*	Our Stock
FY 8000	*h+/− hht1-mRFP:kanMX his7+*::*lacI-GFP lys1+*::*lacO cdc21-c106-HphMx leu1-32 ura4- D18*	This work
FY 8015	*h+* Δ*ctf8*::*kanMX6-Bioneer cdc21-c106*::*HphMx his 3-D1 ura4-D18 Leu1-32 ade6-M210*	This work
FY 8016	*h*- Δ*ctf8*::*kanMX6-Bioneer cdc21-c106*::*HphMx his 3-D1 ura4-D18 Leu1-32 ade6-M210*	This work
FY 8017	*h+* Δ*ctf18*::*kanMX6-Bioneer cdc21-c106*::*HphMx ura4-D18 Leu1-32 ade6-M210*	This work
FY 8018	*h*-Δ*ctf18*::*kanMX6-Bioneer cdc21-c106*::*HphMx ura4-D18 Leu1-32 ade6-M210?*	This work
FY 7689	*h+* Δ*ctf18*::*kanMX6-Bioneer ura4-D18 Leu1-32 ade6-M210?*	Our stock/Bioneer derived
FY 7690	*h*- Δ*ctf18*::*kanMX6-Bioneer ura4-D18 Leu1-32 ade6-M210?*	Our stock/Bioneer derived
FY 8107	*h+/−? cdc20-M10 cdc21-c106*::*kan ura4-D18 ade6- M210 leu1-32 his3-D1 (polε)*	This work
FY 7808	*h+ rad21-K1*::*ura4+ cdc21-c106*::*kan ura4-D18 leu1-32 ade6-M216 his7-366/his 3D-1*	This work
FY 8108	*h+/− ?* Δ*rad22*::*ura4+ cdc21c-106*::*kan ura4-D18 leu1-32 his3-D1 arg3-D4*	This work
FY 5014	*h+ pcn1-K164R*::*ura4 ura4-D18 leu1-32 ade6- M210*	Our stock
FY 5270	*h+ kpa1*Δ::*bleMX6 his4-239 ade6-M26*	Our stock
FY 3124	*h+* Δ*rhp18*::*ura4+ ura4-D18 leu1-32 ade6-704*	Our stock
FY 4415	*h+* Δ*reb1*::*kanMX ade6-M216 ura4-D18 leu1-32*	Our stock
FY 277	*h+ cyc17*::*ura4 ade6-M216 leu1 ura4 cyc17 is allelic to cig2*	Hiroto Okayama
FY 5401	*h+* Δ*rev1*::*ura4+ ura4-D18 his4-239 ade6-M26*	Our stock
FY 4937	*h+ eso1*::*kanMX6 ura4-D18 leu1-32 ade-M210*	Our stock
FY 5142	*h+* Δ*brc1*::*ura4+ ura4-D18 leu1-32 ade6- M210*	Mathew O’Connell
FY 4938	*h+ rev3*::*hphMX6 ura4-D18 leu1-32 ade6- M210*	Our stock
FY 2050	*h+ srs2*::*kan ade6-M210 leu1-32 ura4-D18*	Our stock
FY 5260	*h*- Δ *mms2*::*leu2 leu1-32 his4-239 ade6-M26*	Our stock
FY 5625	*h+* Δ*rad8*::*hphMX leu1-32 ura4-D18 ade6- M216 his3-D1*	Our stock/Bioneer derived
FY 4742	*h- rad11-Cerulean*::*hphMX rad22-YFP-natMX leu1-32 ade6-M210 ura4-D18 (rad11 = ssb1)*	Our stock
FY 4611	*h- chk1HA(ep) ade6-M216 ura4-D18 leu1-32*	Our stock
FY 1163	*h- rad12*::*ura4+ ade6-M210 leu1-32 ura4-D18*	Our stock
FY 3845	*h-leu1-32*::*hENT1-leu1+(pJAH29) his7-366*::*hsv-tk- his7+(pJAH31) ura4-D18 ade6-M216*	Our stock
FY 1257	*h+ mad2D*::*ura4+ ade6-M210 leu1-32 ura4-D18*	Shelly Sazer
FY 5583	*h+* Δ*rif1:kanMX6-Bioneer leu1-32 ura4-D18 ade6- M210 his3-D1*	Our stock/Bioneer derived
FY 3884	*h- exo1*::*ura4 ura4-D18*	Mathew O’Connell
FY 2389	*h- leu1-32 ura4-D18* Δ*rif1*::*ura4+*	Junko Kanoh
FY5555	*h*- Δ*fml1*::*natMX4 ura4-D18 his3-D1 leu1-32*	Our stock
FY 4581	*h- chp1*::*kanMX6-Bioneer leu1-32 ura4-D18 ade6-M216his3-D1*	Our stock
FY4159	*h+* Δ*mus81*::*KanMX*	Our stock
FY1191	*h- mcl1-11 ade6-704 ura4-294 leu1-32 (ts)*	Dwight Williams
FY 1203	*h+ rhp51*::*ura4+ ade6-704 leu1-32 ura4-D18*	Greg Freyer
FY 1318	*h+ rec8*::*ura4+ ura4-D18 leu1-32 ade6-M210*	Our Stock
FY 1159	*h-rad21-K1*::*ura4+ ura4-D18 leu1-32 ade6-M216 his7-366*	Our stock
FY 3588	*h- arg3+*::*ccr1N-mCherry((D817 aa1-275)*::*his5+ ura4-D18 his5D*	Zach Cande/XieTang
FY 3227	*h+* Δ*swi1*::*KanMX ura4-D18 leu1-32 ade6-M210 his3-D1*	Our stock
FY 3228	*h+* Δ*swi3*::*KanMX ura4-D18 leu1-32 ade6-M210 his3-D1*	Our Stock
FY 7995	*h? arg3+*::*ccr1N-mCherry((D817 aa1-275)*::*his5+ rad11-Cerulean*::*hphMX rad22-YFP-kanMX ura4-D18 his5D leu1-32*	This work
FY 5787	*h+ hht1-mRFP:kanMX his7+*::*lacI-GFP lys1+*::*lacO leu1-32 ura4-D18*	Our stock
FY 7653	*h+* Δ*ctf8*::*kanMX6-Bioneer his 3-D1 ura4-D18 Leu1-32 ade6-M210?*	Our stock/Bioneer derived
FY 8017	*h+* Δ*ctf18*::*kanMX6-Bioneer cdc21-c106*::*HphMx ura4-D18 Leu1-32 ade6-M210*	This work/Bioneer derived
FY 8110	*h?psf2-209 cdc21-c106*::*Kan ura4-D18leu1-32 ade6-M216*	This work
FY 8111	*h? rad35-271 cdc21-c106*::*Kan ura4-D18 leu1-32 ade6-M216*	This work
FY 2711	*h+ psf2-209 ura4-D18 ade6-M216 leu1-32*	Our stock
FY 3999	*h+ rad35-271 allelic to dfp1*	Our stock
FY 8197	*h- pol1-1 cdc21-c106*::*Kan ura4-D18 leu1-32 ade6-M210 his3-D1*	This work
FY 1110	*h+ pol1-1 ura4-D18 leu1-32 ade6-M210*	Our stock

### Serial dilution assays and relative viability

For serial dilutions, cell cultures were grown in 5 ml of YES from a single colony at 25° overnight, to midexponential phase. Cells were counted and fivefold serial dilutions were spotted onto plates to assess drug or temperature sensitivity. Drug plates were allowed to grow for 3–5 d at 25° before scanning on a flatbed scanner. The experiments were repeated at least twice. For relative viability, cells at OD_595_ ∼0.3 were treated with 0.01% MMS for 4–6 hr or shifted to 36° for 4–6 hr (as indicated in the figure legend). Samples were collected every 2 hr and fixed in 70% ethanol for Flow Cytometry (FACS) and DNA staining with DAPI. Serial dilutions of equal volumes were plated at selected time points and allowed to grow at 25° for 5 d before counting viable colonies.

### Protein extractions

Western blot analysis was performed using cultures grown to early log phase (OD_595_ ∼0.3) in YES at 25°. Cultures were split into equal volumes and treated with 0.01% MMS or left untreated for 4 hr at 25°. 10 × stop buffer containing 2% sodium azide was added and cultures were incubated on ice for 10 min before harvesting the cells. Cells were subsequently washed twice with 1 × PBS and whole cell proteins were extracted using trichloric acid (TCA) (Foiani *et al.* 1994). The extractions were quantified using a Pierce BCA Kit. 80–100 μg of protein was loaded on 8% SDS-PAGE gels. Primary antibody for Chk1HA (16B12 anti-HA; Covance or anti-HA; Abcam) were used in 1:1500 dilutions overnight at 4°. Mcm4 protein levels were detected with antibody purified from rabbit serum 5898 diluted 1:3000 (Sherman *et al.* 1998) incubated at 4° overnight. After washing with PBST, anti-mouse-IgG-HRP secondary antibody sigma) was used to detect HA in a 1:5000 dilution, while a 1:5000 dilution of anti-rabbit-HRP (BD Biosciences) incubated for 1 hr at room temperature was used in Mcm4 detection. 1:1500 PCNA anti-mouse (Santa Cruz) was used as the loading control.

### Pulse field gel electrophoresis (PFGE)

PFGE was performed to separate full-length chromosomes using a BioRad Chef II Pulse Field Machine. 50 ml cultures grown to early log phase (OD_595_ ∼0.3–0.4) were shifted to 36° for 4 hr and released to permissive temperature 25° for 2 hr. For the MMS treatment, the cells were treated with 0.01% MMS for 4 hr and released to media lacking MMS for 2 hr for recovery after washing out the drug from the cultures. Cells were treated with 10 × stop buffer containing 2% sodium azide and placed on ice for 5 min before harvesting the cells. Harvested cells were washed with 1 × PBS and CSE buffer (20 mM Citric Acid, 20 mM Na_2_HPO_4_, 40 mM EDTA, and 1.2 M sorbitol; at pH 5.6, sterilized, and stored at room temperature). Each culture was digested with 0.2 mg/ml 20T Zymolase and 0.45 mg/ml lysing enzyme (Sigma) in CSE. Digested cells were used to prepare plugs that were resuspended in 1 × TSE (10 mM Tris pH 7.5, 45 mM EDTA pH 8.0, and 0.9 M Sorbitol). Plugs were treated with 5 ml of 1 mg/ml proteinase K Sarkosyl/EDTA at 55° for 48 hr (1% Sarkosyl and 0.5 M EDTA pH 9.5). The buffer was changed after 24 hr of incubation and washed four times (30 min each) with 1 × TE. Plugs were washed with 1 × TAE prior to running the gels. Gels were run for 48 hr using 2 V/cm, 1200–1800-sec switch time, and a 106° angle. DNA was visualized via ethidium bromide staining.

### FACS

FACS was performed as described in (Sabatinos and Forsburg 2015). Briefly, cells were fixed in 70% ice cold ethanol, washed with 50 mM sodium citrate, and resuspended in 50 mM sodium citrate with 0.1 mg/ml RNAse. Samples were next stained with 1 µM Sytox Green (Invitrogen) in 50 mM sodium citrate, and sonicated at 20% amplitude for 5 sec. Samples were analyzed by running on a Becton Dickinson FACScan flow cytometer.

### Microscopy

Cultures were grown in EMM supplement with ammonium chloride. Agar pads were prepared as described in Green *et al.* (2015). Images of live cells were acquired with a DeltaVision Core (Applied Precision, Issaquah, WA) microscope using a 60 × N.A. 1.4 PlanApo objective lens and a 12-bit Photometrics CoolSnap HQII CCD. The system x-y pixel size is 0.109 µm. SoftWoRx v4.1 (Applied Precision, Issaquah, WA) software was used at acquisition. The image acquisition consisted of 13 Z-stacks with 0.5 µm for visualizing Rad11 and Rad52 foci at 36° and MMS. Cells were visualized at the asynchronous stage; 4 hr posttreatment and 2 hr postrelease from the treatment. Movies were captured to look at the replication dynamics in real time. Eighteen Z-stacks with 0.5 µm were acquired 10 min apart for the length of the experiment. The temperature was controlled at 25° if not specified. For still imaging, CFP was excited and detected with an (ex)438/24, (em)470/24 filter set and a 0.5 sec exposure excitation intensity attenuated to 10%; and YFP was excited and detected with an (ex)513/17, (em)559/38 filter set and a 0.5 sec exposure excitation intensity attenuated to 32%. Suitable polychroic mirrors were used. Ten 0.5 µm serial z-sections were captured. 3-D stacks were deconvolved with manufacturer-provided OTFs using a constrained iterative algorithm, images were maximum intensity projected for presentation. Images were contrast adjusted using a histogram stretch with an equivalent scale and γ for comparability.

### Data availability

Strains are available upon request. The authors state that all data necessary for confirming the conclusions presented in the article are represented fully within the article.

## Results

### Identification of an MMS-sensitive allele of mcm4^+^

Mutants with defects in replisome components often show sensitivity to DNA damaging agents, but not all mutants are sensitive to all drugs. For example, in a recent study, we showed that cells deleted for nonessential helicases have distinct patterns of genotoxin sensitivity that establish a fingerprint for their roles in DNA replication and repair (Ding and Forsburg 2014). Here, we analyzed a panel of strains with different mutations in the essential *mcm4^+^* gene for their sensitivity to different damaging agents, including: HU, which depletes nucleotide pools and causes fork stalling (Thelander and Reichard 1979); MMS, which is an alkylating agent that generates diverse lesions that block DNA polymerase (Lundin *et al.* 2005); and camptothecin (CPT), a topoisomerase inhibitor that leads to S-phase specific double strand breaks (Liu *et al.* 2000).

**Figure 1:**
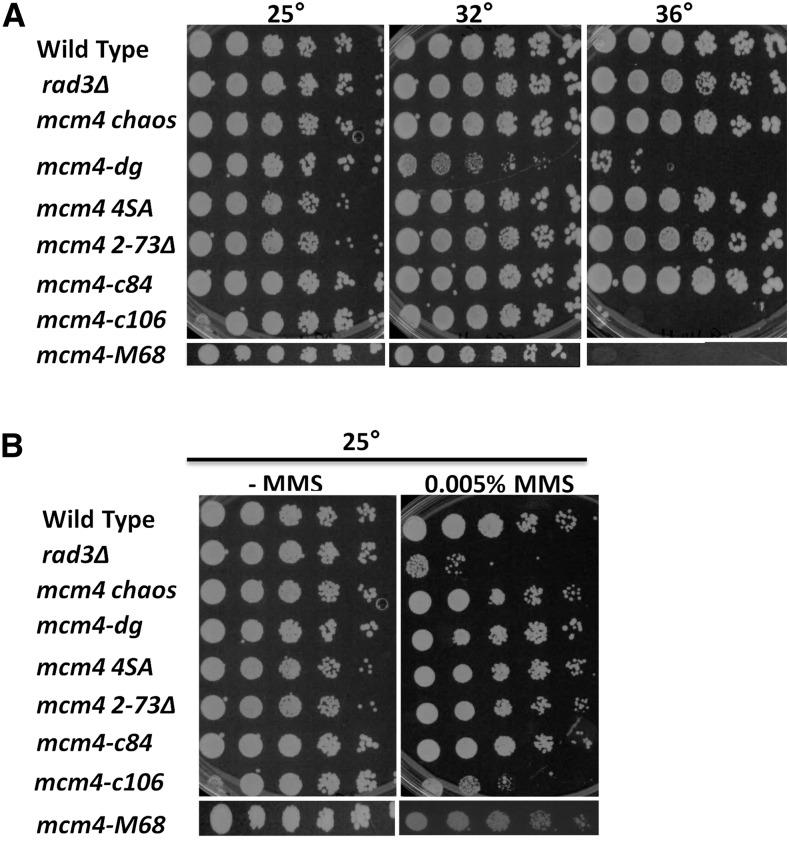
Viability of *mcm4* mutants at 36° and in MMS. (A) Temperature sensitivity evaluated by 1:5 serially diluted cultures plated on YES (rich media) and grown at the indicated temperatures. Wild-type (FY 528), *rad3*∆ (FY1106), *mcm4 chaos* (FY 3664), *mcm4-dg* (FY 3395), *mcm4-4SA* (FY5251), *mcm4 2-73*∆ (FY 5688), *mcm4-c84* (FY4310), *mcm4-c10*6 (FY 4311), and *mcm4-M68* (FY784). (B) MMS sensitivity evaluated by 1:5 serially diluted cultures plated on YES (rich media) as control and 0.005% MMS at 25°. MMS, methyl methanesulfonate; YES, yeast extract + supplements.

We examined the known temperature-sensitive alleles *mcm4-M68*, *mcm4 dg*, and *mcm4-c106* (Nasmyth and Nurse 1981; Lindner *et al.* 2002; Nitani *et al.* 2008). The remaining mutants that we tested included a deletion of the N-terminal residues 2–73, a single point mutation (F346I) corresponding to the *chaos* allele in mouse (J. P. Yuan and S. L. Forsburg, unpublished data; Shima *et al.* 2007), and *mcm4-4SA*, which contains mutations in putative damage-specific phosphorylation sites S30A, S38A, S81A, and T95A (Figure 1A). As observed previously, some *mcm4* mutants show sensitivity to HU, including the temperature-sensitive *degron* allele *mcm4-dg*, (Supplemental Material, Figure S1A) and the C-terminal truncation alleles *mcm4-c84* and *mcm4-c106* (Nitani *et al.* 2008). We did not observe CPT sensitivity in any of the strains (Figure S1B). Unexpectedly, however, we observed that the temperature-sensitive *mcm4-c106* truncation is also sensitive to MMS exposure at the permissive growth temperature, which is not seen for any other *mcm4* alleles **(**Figure 1B). Importantly, temperature-sensitive and MMS-sensitive phenotypes were not observed for the *mcm4-c84* truncation, which contains a shorter truncation (Nitani *et al.* 2008). These results indicate that a larger C-terminus of Mcm4 is necessary for a proper response to MMS.

In general, sensitivity to MMS is observed in strains defective in checkpoint response or repair, and mutations that disrupt a distinct subset of replisome components. These include mutations affecting the fork protection complex (FPC) proteins Swi1 and Swi3, and the MCM kinase Hsk1/DDK that interacts with FPC (Memisoglu and Samson 2000; Noguchi *et al.* 2003, 2004; Kumar and Huberman 2004; Sommariva *et al.* 2005; Dolan *et al.* 2010). Given that *mcm4-c106* shows sensitivity to MMS as well as to higher temperatures, we investigated both these phenotypes.

### mcm4-c106 cells have a unique replication phenotype

**Figure 2:**
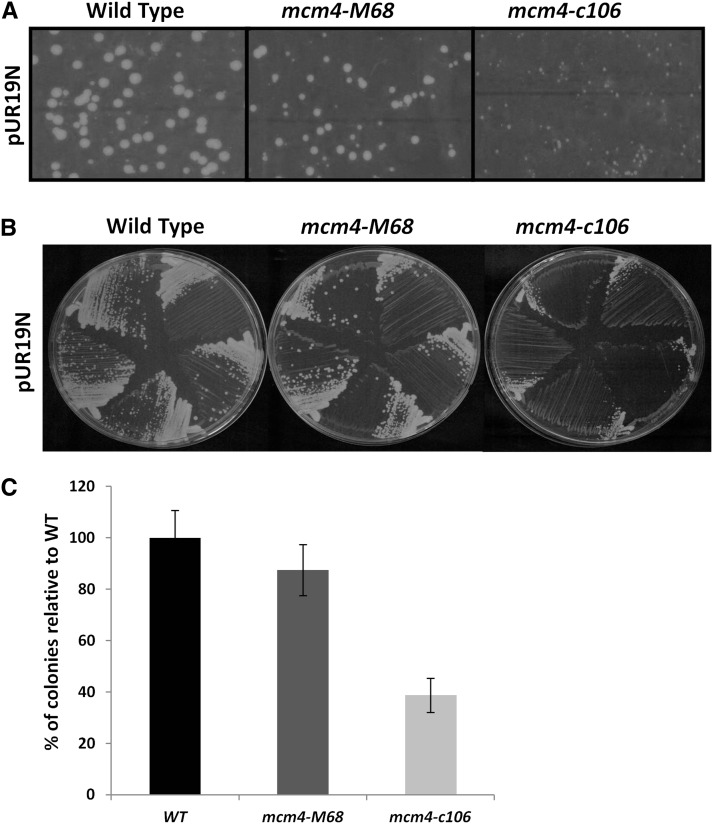
*mcm4-c106* has a defect in replication at permissive temperature. (A) WT (FY 528), *mcm4-M68* (FY784), and *mcm4-c10*6 (FY 4311) strains transformed with pUR19N plasmid plated on media lacking uracil. Colonies observed after 7 d of growth at 25°. (B) Colonies isolated from transformation (A), streaked to single colonies on media lacking uracil, and grown at 25° for 5 d. (C) Percentage of colonies observed posttransformation of 200 ng/µl pUR19N plasmid relative to the wild type. Error bars represent 95% C.I. of the mean. WT, wild-type.

There is no obvious difference in growth rate between wild-type and *mcm4-c106* cells at permissive temperature (Figure 1A). To assess whether there is a subtle replication defect, we performed a classic minichromosome maintenance assay (Tye 1999). We transformed the wild-type, *mcm4-M68*, and *mcm4-c106* strains with a plasmid (pUR19N; Barbet *et al.* 1992) containing a single copy of *Schizosaccharomyces pombe ars1* and compared the number of transformants/µg DNA (transformation efficiency) and plasmid stability (colony size). We found that both the number of transformed cells and the size of the colonies were reduced in *mcm4-c106* compared to either *mcm4-M68* or the wild type at permissive temperature (Figure 2). Additionally, we examined a plasmid with an additional *ars* (pDblet), which is intrinsically more stable (Brun *et al.* 1995). These transformants were larger than when transformed with a single *ars* plasmid (data not shown). These data suggest that *mcm4-c106* suffers a defect in replication, even at permissive temperature.

**Figure 3:**
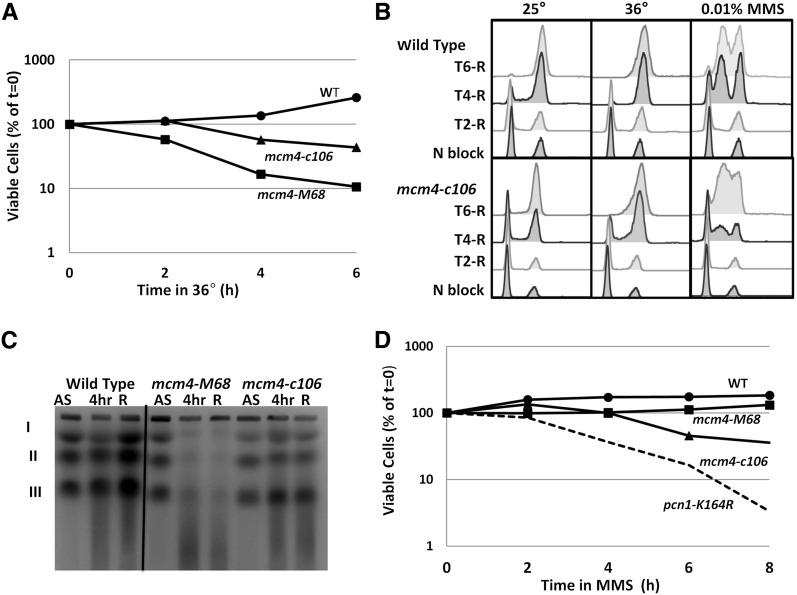
Replication dynamics in *mcm4-c106* at 36° and MMS. (A) Relative viability of cultures during incubation at 36°. The indicated cultures were plated at 25° on YES, and viability was compared to the starting culture. (B) Bulk DNA content measured by flow cytometry of Sytox Green labeled cells. Cells were synchronized in G1 by nitrogen starvation and released to 25°, 36°, and 0.01% MMS nitrogen-containing medium. WT (FY261), *mcm4-c106* (FY 4311). The indicated times correspond to the time after release. (C) Pulsed field gel electrophoresis (PFGE) analysis in each genotype in untreated asynchronous (AS) cells, after 4 hr 36° (4 hr) and after 2hr release to 25° (R). WT (FY 528), *mcm4-M68* (FY784), and *mcm4-c106* (FY 4311). *S. pombe* chromosomes are indicated on the left. (D) Relative viability of cultures during incubation with 0.01% MMS. The indicated cultures were plated at 25° on YES plates and the viability was compared to the starting culture. MMS, methyl methanesulfonate; WT, wild-type; YES, yeast extract + supplements.

At 36°, the canonical *mcm4*-M68 allele loses viability rapidly, with signs of DNA damage (Liang and Forsburg 2001**;** Bailis *et al.* 2008**;** Sabatinos *et al.* 2015**). We** examined the relative viability of *mcm4-c106* following a shift to the restrictive temperature, and found that the loss of viability was more modest compared to *mcm4-M68* (Figure 3A). We examined DNA accumulation using flow cytometry on cells that were arrested in G1 by nitrogen starvation, and released to the permissive temperature (25°) and the restrictive temperature (36°) (Figure 3B). We observed DNA accumulation to approximately 2C DNA content in both wild-type and *mcm4-c106* cells, even at restrictive (36°) and nonrestrictive (25°) temperatures. This is similar to observations for the original *mcm4-M68* temperature allele, which has a late S phase arrest (Nasmyth and Nurse 1981; Coxon *et al.* 1992; Bailis *et al.* 2008; Sabatinos *et al.* 2015).

However, the chromosome profiles observed in PFGE were strikingly different between these two *mcm4* alleles. Typically, the chromosomes from cells with replication defects do not migrate normally at restrictive temperature, either due to unresolved replication or recombination intermediates that preclude migration, or due to chromosome breakage (*e.g.*, Liang and Forsburg 2001; Waseem *et al.* 1992). Thus, as seen previously, the *mcm4-M68* chromosomes do not migrate at their normal position during a 36° temperature shift or upon release to 25°, but are replaced by a smear (Figure 3C, middle lanes). This is consistent with unresolved replication intermediates retarding gel migration, and the accumulation of double strand breaks as reported previously (Liang and Forsburg 2001; Bailis *et al.* 2008; Sabatinos *et al.* 2015). In contrast, *mcm4-c106* showed intact chromosomes under all conditions (Figure 3C, right lanes). This, along with the maintenance of viability and the ability to recover from temperature arrest, suggests that the nature of the temperature-sensitive defect in *mcm4-c106* is different from that of the well-studied *mcm4-M68*.

We observed loss of viability of *mcm4-c106* cells treated with MMS (Figure 3D) consistent with the MMS sensitivity observed in plate assays (Figure 1B). The loss of viability was relatively modest compared to a repair-defective allele of PCNA, *pcn1-K164R* (Frampton 2006). During MMS treatment in liquid culture at the permissive temperature, both wild-type and *mcm4-c106* mutants showed an S phase delay, as indicated by the intermediate peak that is observed in the FACS profiles (Figure 3B). In both wild-type and *mcm4-c106* cells treated with MMS and following release, we observed little if any migration of intact chromosomes into a PFGE gel **(**Figure S2).

**Figure 4:**
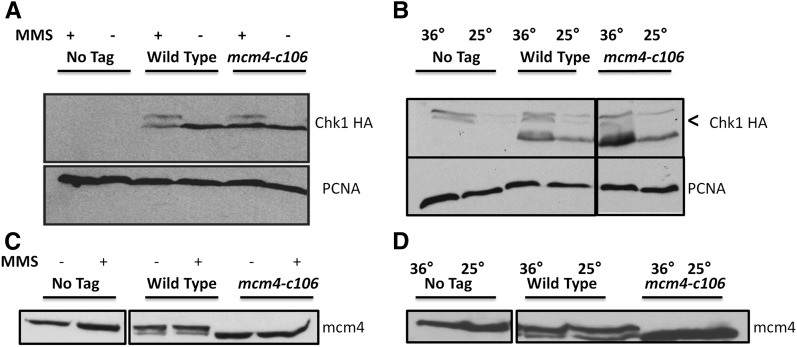
Mcm4 protein levels and Chk1 phosphorylation in response to MMS and 36°. (A) Evidence for Chk1 activation following 0.01% MMS for 4 hr. Chk1 mobility in SDS-PAGE was used as a proxy for phosphorylation. Lanes 1 and 2, *mcm4-c106* (FY 4311); lanes 3 and 4, *chk1-HA* (FY 4611); lanes 5 and 6 *mcm4-c106 chk1-HA (*FY6281). Arrow indicates phosphoshift. (B) Activation of Chk1 following 4 hr incubation at 36°. Lanes 1 and 2, *mcm4-c106* (FY 4311); lanes 3 and 4, *chk1-HA* (FY 4611); lanes 5 and 6, *mcm4-c106 chk1HA (*FY6281). (C) Mcm4 protein levels following MMS treatment. (D) Mcm4 protein levels following incubation at 36°. MMS, methyl methanesulfonate; PCNA, proliferating cell nuclear antigen.

Finally, we examined Mcm4 protein levels in the mutant. Loss of Mcm4 protein has been correlated with genomic instability (Liang and Forsburg 2001; Bailis *et al.* 2008; Sabatinos *et al.* 2015). However, we saw no change in Mcm4 protein levels during MMS treatment or at 36° in *mcm4-c106* (Figure 4, C and D), suggesting that its temperature sensitivity and MMS phenotypes are not related to protein stability.

### Chromosome segregation is normal in mcm4-c106

Recently, we showed that *mcm4 dg* mutants undergo division despite their replication defects, and this is accompanied by aberrant nuclear division, abnormal chromosome segregation, and reduced viability (Sabatinos *et al.* 2015). In contrast, we saw no evidence for abnormal mitosis in *mcm4-c106* cells. We determined segregation of chromosome I, using a *lacI*-GFP fusion in a strain with a *lacO* array at centromere I to generate a centromere proximal signal (Nabeshima *et al.* 1998). We observed no evidence for lagging chromosomes, or chromosome mis-segregation, indicating no substantial mitotic defects in *mcm4-c106* (Figure S3).

### mcm4-c106 requires an intact damage checkpoint

The *mcm4-c106* cells elongate following treatment at 36° or in MMS, which suggests successful activation of the damage checkpoint. We verified this by monitoring the checkpoint kinase Chk1, which undergoes an activating phosphorylation that results in a mobility shift in SDS-PAGE (Walworth and Bernards 1996). We observed a shift in Chk1 in both wild-type and *mcm4-c106* cells treated with MMS (Figure 4A), and in wild-type and *mcm4-c106* cells at the restrictive temperature (Figure 4B), consistent with successful activation of Chk1 under both conditions in the mutant.

We observed no evident Chk1 phosphorylation in asynchronously growing cultures at 25° in the absence of treatment (Figure 4, A and B). Despite this, we found that double mutants between the *mcm4-c106* and either *rad3*∆ or *chk1*∆ were inviable. However, double mutants with the S phase checkpoint mutant *cds1*∆ were viable (Table 2). We conclude that, even though we do not observe shifted mobility of Chk1 at permissive temperature, there is sufficient stress even in unperturbed *mcm4-c106* cells to cause them to depend upon the damage checkpoint for viability.

**Table 2:** Sensitivity to MMS of double mutants of genes involved in different aspects of the cell cycle

Category	Mutant	Function	Phenotype with c106*^a^*	MMS Phenotype*^b^*
Checkpoint	*cds1*Δ	Kinase; S phase/replication checkpoint	Viable	>*mcm4-c106*
Checkpoint	*chk1*Δ	Kinase; G2/damage checkpoint	Synthetic lethal	ND
Checkpoint	*mad2*Δ	Spindle checkpoint	Viable	=*mcm4-c106*
Checkpoint	*rad26*Δ	Checkpoint protein	Synthetic lethal	ND
Checkpoint	*rad3*Δ	Kinase	Synthetic lethal	ND
Cohesion	*chl1*Δ	Helicase	Viable	> *mcm4-c106*
Cohesion	*rad21-K1*	Cohesin protein	Viable	= *rad21-K1*
Replication	*mcl1-1 ts*	Part of the FPC/cohesion	Sick	ND
Replication-FPC	*mrc1*Δ	Replication mutants/FPC	Synthetic lethal	ND
Replication-FPC	*swi1*Δ	Replication mutants/FPC	Synthetic lethal	ND
Replication-FPC	*swi3*Δ	Replication mutants/FPC	Synthetic lethal	ND
Replication	*hsk1-1312*	DDK kinase	Synthetic lethal	ND
Replication	*dfp1-r35*	DDK kinase	Viable/sick	ND
Replication, genome stability	*rif1*Δ	Rif1 antagonist of DDK	Viable	rescued
Genome stability	*brc1*Δ	Genome stability	Viable	*mcm4-c106*
Replication clamp loader	*ctf8*Δ	Cohesion-specific clamp loader	Viable < temp	*= mcm4-c106*
Replication clamp loader	*ctf18*Δ	Cohesion-specific clamp loader	Viable < temp	*= mcm4-c106*
Genome stability	*fml1*Δ	Helicase; genome stability	Viable	*=fml1*Δ
Genome stability	*mus81*Δ	Holiday junction resolvase	Sick	=*mus81*
Genome stability	*rqh1*Δ	Helicase; recombination antagonist	Viable	> *mcm4-c106*
Genome stability	*srs2*Δ	Helicase; recombination regulator	Viable	>*mcm4-c106*
Repair	*eso1*-Δ*eta*	Eso1- Polη fusion; deletes polymerase domain. Error prone repair	Viable	≤*mcm4c106*
Repair	*exo1*Δ	Exonuclease I	Viable	= *exo1*Δ
Repair	*mms2*Δ	Ubiquitin ligase; error free repair	Viable	> *mms2*Δ
Repair	*pcn1-K164R*	PCNA; ubiquitin site mutant	Viable	= *pcn1-K164R*
Repair	*polk*Δ	*Pol*κ; error prone repair	Viable	*>mcm4-c106*
Repair	*rad51*Δ	Homologous recombination	Viable	=*rad51*Δ
Repair	*rad8*Δ	Ubiquitin ligase-helicase; error free repair	Viable	*>rad8*Δ
Repair	*rev1*Δ	Deoxycytidyl transferase; error prone repair	Viable	≥ *mcm4-c106*
Repair	*rev3*Δ	*Pol*ζ Error prone repair	Viable	*<mcm4-c106*
Repair	*rhp18*Δ	PCNA ubiquitin ligase	Viable	= *rhp18*Δ
Repair	*ubc13*Δ	Ubiquitin ligase; error free	Viable	> *ubc13*Δ
	*chp1*Δ	Heterochromatin protein	Viable	=*mcm4-c106*
	*cig2*Δ*/_cyc17*	S phase cyclin	Viable	≤ *mcm4-c106*
	*reb1*Δ	Transcription termination	Viable < temp	=*mcm4-c106*
	*swi6*Δ	Heterochromatin protein	Viable	= *mcm4-c106*
	*rad22*Δ		Viable/slow growing	ND
	*cdc20- M10(pol*ε*)*		Viable	ND
	*cdc6- 23(pol*δ*)*		Synthetic lethal	NA
	*psf2ts*	Component of the GINS replication complex	Viable	*>mcm4-c106*
	*pol1-1(pol*α*)*		Viable	ND
	*rad50*Δ		Synthetic lethal	NA

Double mutant phenotypes between *mcm4-c106* and other mutants in the indicated classes. MMS, methyl methanesulfonate; ND, Not Determined; FPC, fork protection complex; DDK, Dbf4-dependent kinase; PCNA, proliferating cell nuclear antigen; NA, Not Applicable.

a Phenotype: viable means no change in temperature sensitivity. Synthetic lethal is dead at temperatures. Sick shows reduced growth rate at all temperatures. <temp has reduced maximum growth temperature.

b MMS phenotype is determined relative to the most sensitive parent (indicated) being > (greater sensitivity), < (less sensitive), or = (equal sensitivity).

### Repair foci accumulate in mcm4-c106

**Figure 5:**
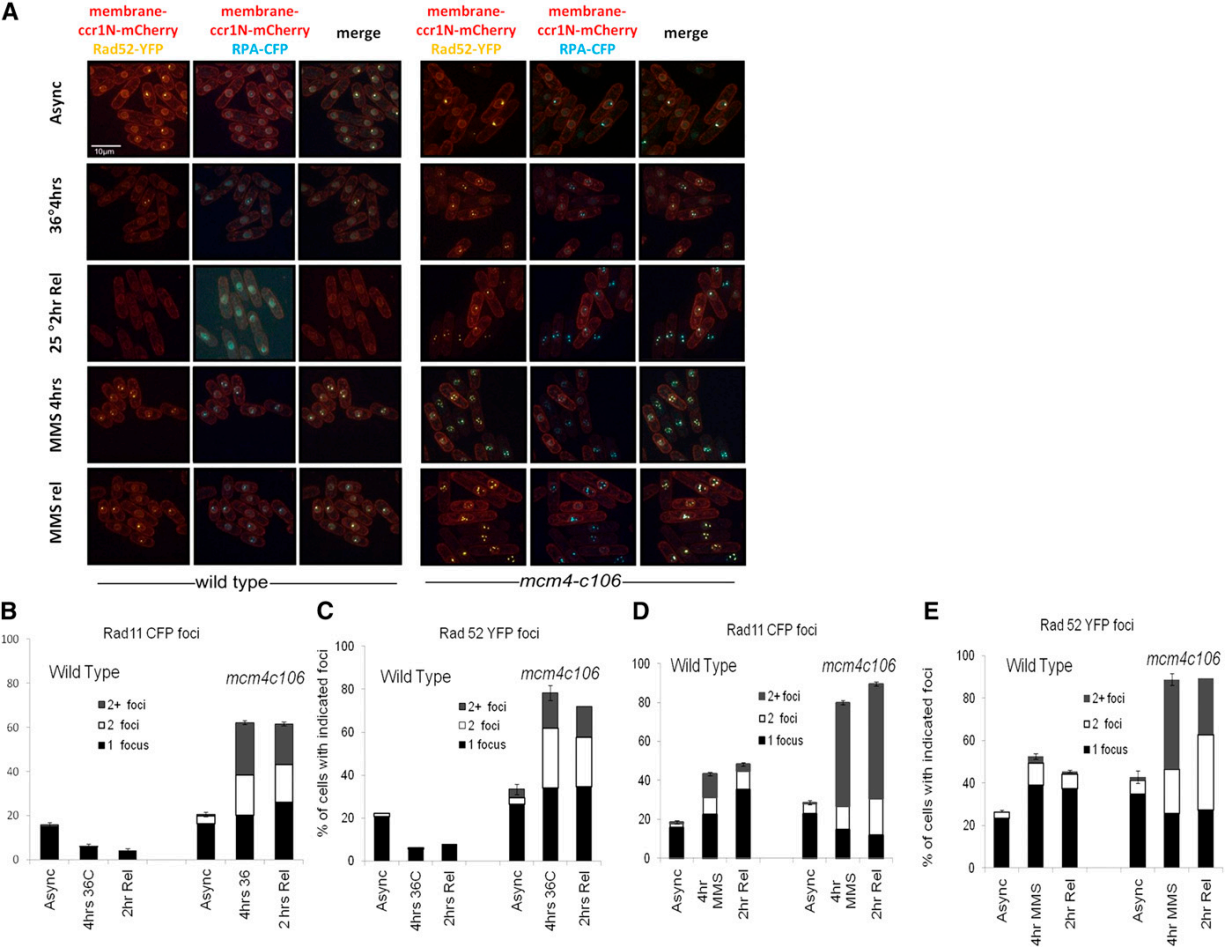
Accumulation of repair foci in *mcm4-c106* in response to MMS and temperature. (A) Membrane (ccr1N-mCherry), RPA-CFP (blue), and Rad52-YFP (yellow) focus patterns during treatment at restrictive temperature (36°) and release. Multiple small foci were observed with the *mcm4-c106* that remained after release. RPA and Rad52 focus patterns during treatment with 0.01% MMS for 4 hr and release for 2 hr. Multiple small foci were observed during treatment and release in the *mcm4-c106* compared to the wild type. Scale 10 µm. (B) Quantification of Rad11 foci of wild-type and *mcm4-c106* during 36° treatment and release. (C) Quantification of Rad52 foci of wild-type and *mcm4-c106* during 36° treatment and release. (D) Quantification of Rad11 foci of wild-type and *mcm4-c106* during 4 hr MMS treatment and release. (E) Quantification of Rad52 foci of wild-type and *mcm4-c106* during 4 hr MMS treatment and release. Two or more independent experiments were pooled and a 95% C.I. was calculated. Async, asynchronous; MMS, methyl methanesulfonate.

Previously, we examined replication stress by examining the accumulation of repair foci corresponding to the single strand DNA binding protein RPA (labeled with CFP), or the recombination protein Rad52 (labeled with YFP), and have observed differences in their distribution, pattern, and intensity in different conditions (Bailis *et al.* 2008; Sabatinos *et al.* 2012, 2015). There are dramatically different phenotypes between two different temperature-sensitive alleles. *mcm4-M68* forms multiple small foci and robustly arrests division at permissive temperature, while *mcm4-dg* forms a single large megafocus and undergoes continued division (Bailis *et al.* 2008; Sabatinos *et al.* 2015). Therefore, we examined repair foci fluorescence in both wild-type and *mcm4-c106* cells either shifted or released from 36°, or from MMS (Figure 5).

At 25°, *mcm4-c106* shows a modest increase in cells with both foci compared to the wild type, consistent with an increased basal level of stress (Figure 5, A–C). Following a shift to 36°, *mcm4-c106* accumulated numerous small foci of RPA-CFP and Rad52-YFP and these remain after 2 hr of release, whereas the foci in wild-type cells decline by 4 hr at 36° (Figure 5, A–C) (Sabatinos *et al.* 2015). The *mcm4-c106* cells did not divide in the first 2 hr following release. This suggests multiple dispersed damage sites, similar to *mcm4-M68*.

In wild-type cells during a 4 hr treatment with 0.01% MMS or at 2 hr after release from MMS, there is modest increase in cells with RPA or Rad52 foci (Figure 5, A, D, and E). This increase is measured from approximately 20% in untreated cells to about 50% in treated cells, but most of these have just one or two foci. In contrast, while *mcm4-c106* cells have similar overall levels of focus formation in untreated cells, up to 80% of the cells have at least one focus in treated cells, and a strikingly large fraction contains multiple bright signals, which persist through the period of release. The majority of the RPA foci observed overlap with Rad52 foci (Figure 5, A, D, and E). Therefore, there is evidence for constitutive repair foci in *mcm4-c106* cells and these are dramatically increased upon exposure to MMS.

### rif1∆ rescues mcm4-c106 MMS phenotype

One response to replication stress is to activate dormant origins (reviewed in Alver *et al.* 2014). The *mcm6-S1* mutant, which affects another subunit of the MCM complex, is the only other MCM allele that displays MMS sensitivity (Maki *et al.* 2011). Deletion of the S-phase cyclin *cig2* rescues this sensitivity, presumably by delaying G1/S phase and allowing additional licensing of origins (Maki *et al.* 2011). Therefore, we examined a double mutant of *cig2*∆ *mcm4-c106*. In contrast to the results reported for *mcm6-S1*, we observed only a very slight suppression of MMS sensitivity (Figure S4).

**Figure 6:**
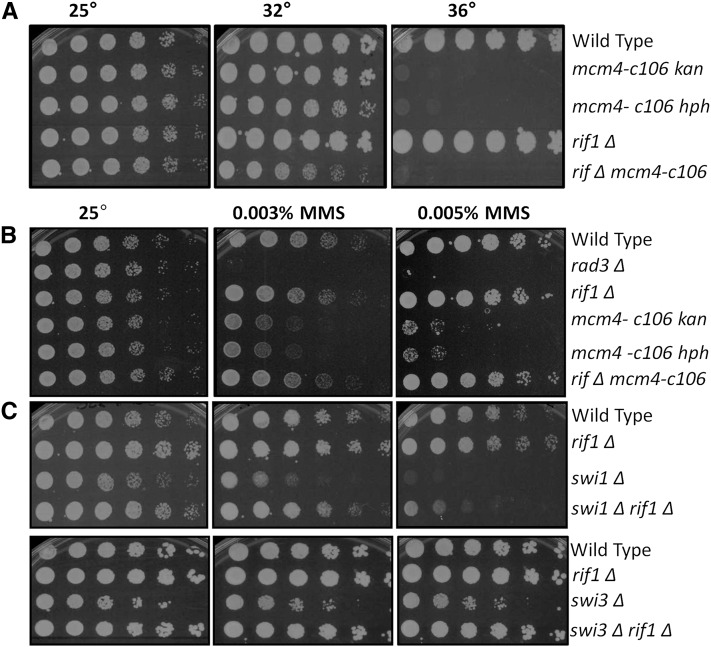
*rif1*∆ rescues the *mcm4-c106* MMS phenotype. (A) Temperature sensitivity evaluated by strains grown overnight at 25°, 1:5 serially diluted, and plated on YES (rich media) at 25° as control, and 32° and 36° to observe the temperature effect. (B) and (C) MMS sensitivity evaluated by strains grown overnight at 25°, 1:5 serially diluted, and plated on YES (rich media) as control, and 0.003% and 0.005% MMS at 25°. MMS, methyl methanesulfonate; YES, yeast extract + supplements.

Unexpectedly, a double mutant *rif1*∆ *mcm4-c106* showed a dramatic rescue of MMS sensitivity (Figure 6B). Rif1 has recently been identified as an antagonist of DDK kinase-mediated phosphorylation of MCM, and regulates timing of origin firing (Hayano *et al.* 2012; Yamazaki *et al.* 2013; Davé *et al.* 2014; Hiraga *et al.* 2014; Mattarocci *et al.* 2014). Despite this dramatic rescue of the MMS phenotype, however, *rif1*∆ does not rescue the temperature sensitivity of *mcm4-c106* (Figure 6A).

### Mcm4-c106 requires fork protection complex for viability

There is a central replisome scaffold that links the leading and lagging strand polymerases and the MCM helicase, including Mcl1(ScCtf4), Mrc1, the fork protection complex (FPC) Swi1(ScTof1), and Swi3(ScCsm3) (reviewed in Aze *et al.* 2013; Leman and Noguchi 2012; Errico and Costanzo 2012). Mutants defective in these nonessential proteins are all sensitive to MMS, indicating that robust coupling of the helicase and polymerase is required for proper response to alkylation stress. We observed that, similar to *mcm4-c106*, the MMS sensitivity associated with *swi1*∆ and *swi3*∆ is suppressed in *rif1*∆ double mutants (Figure 6C and Table 2), suggesting a related function for the C-terminus and Swi1 and Swi3. Therefore, we tested epistasis between *mcm4-c106* and FPC components. We found that double mutants between *mcm4-c106* and *swi1*∆, *swi3*∆, or *mrc1*∆ are all inviable, even at 25°. Deletion of *rif1*∆ did not rescue the inviability of *swi1*∆ *mcm4-c106* or *swi3*∆ *mcm4-c106* strains. A double mutant between the *mcl1-1* temperature-sensitive strain (Sc CTF4) (Williams and McIntosh 2002) and *mcm4-c106* was isolated, but grew so poorly that it was impossible to assess its MMS sensitivity (Table 2).

The DDK kinase Hsk1 (ScCdc7) is essential for DNA replication, in part due to its phosphorylation of MCM proteins (Masai *et al.* 2000, 2006; Sheu and Stillman 2006). It also interacts with the fork protection complex (Sommariva *et al.* 2005; Matsumoto *et al.* 2005) and antagonizes Rif1 (Hayano *et al.* 2012; Davé *et al.* 2014). Previously, we showed that the temperature-sensitive mutant *hsk1-1312* is sensitive to MMS and that wild-type Hsk1/DDK persists on the chromatin during MMS treatment, which depends upon the regulatory subunit Dfp1 (Dolan *et al.* 2010). Consistent with Hsk1 working in concert with FPC, we observed that the double mutants of *mcm4-c106 hsk1 1312* and *mcm4-c106 dfp-r35* formed microcolonies that could not be propagated. Thus, FPC and Hsk1, which are important for the MMS response, are also essential in the absence of the Mcm4 C-terminus.

**Figure 7:**
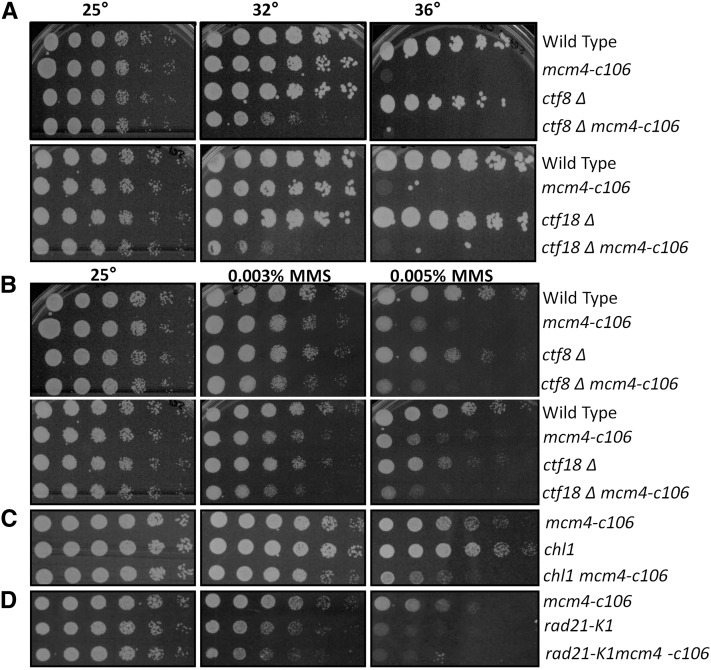
*mcm4-c106* interactions with alternative replication factor C (RFC). (A) *mcm4-c106* combined with RFC^Ctf18^∆ and RFC^Ctf8^∆. Representative response to temperature was assayed by serial dilutions. Strains were grown overnight at 25°, 1:5 serially diluted, and plated on YES (rich media) as the control, and 32° and 36° to observe the temperature effect. (B) Representative response to MMS assessed by serial diluted samples plated on the indicated concentrations of MMS. (C) *chl1*∆ *mcm4-c106* and (D) cohesion subunit *rad21-K1 mcm4-c106* effects on MMS. MMS, methyl methanesulfonate; YES, yeast extract + supplements.

The Ctf18 protein is part of an alternative replication factor C complex RFC clamp loader (Mayer *et al.* 2001; Hanna *et al.* 2001) that is associated with DNA polε (García-Rodríguez *et al.* 2015). In budding yeast and humans, Ctf18 associates with two additional subunits, Dcc1 and Ctf8, to form a heptameric complex that has been shown to have a role in sister chromatid cohesion (Mayer *et al.* 2001; Xu *et al.* 2007; Gellon *et al.* 2011) and a role in facilitating genomic stability (Gellon *et al.* 2011); this complex is also required for the replication checkpoint (Kubota *et al.* 2011). Additionally, in fission yeast, *ctf18*∆ is lethal with *swi1*∆ and *swi3*∆ (Ansbach *et al.* 2008). We find that double mutants *mcm4-c106 ctf18*∆ and *mcm4-c106 ctf8*∆ are viable, but with a modestly reduced permissive temperature (32°) (Figure 7A). They show a little change in MMS sensitivity relative to their parents (Figure 7B and Table 2). This suggests that the Ctf18 complex may be part of a common epistasis group with the Mcm4 C-terminus.

Chl1 is a helicase linked to the lagging strand that is a high copy suppressor of *swi1*∆ damage sensitivity (Ansbach *et al.* 2008), and it further has a role in sister chromatid cohesion in mitosis (Petronczki *et al.* 2004). *chl1*∆ is lethal when combined with *ctf18*∆ (Ansbach *et al.* 2008). Double mutants with *mcm4-c106* and *chl1*∆ show increased sensitivity to MMS compared to their parents, but no effects on temperature (Figure 7C).

*S. pombe* Swi1 and Swi3, Ctf18 complex, and Hsk1 have all been linked to defects in chromosome cohesion (Bailis *et al.* 2003; Ansbach *et al.* 2008; Rapp *et al.* 2010). A temperature-sensitive mutation affecting the cohesin subunit *rad21-K1* combined with *ctf18*∆ shows increased sensitivity to MMS compared to the parents (Ansbach *et al.* 2008). Therefore, we examined a double mutant *mcm4-c106 rad21-K1*. This strain is viable and shows a similar MMS sensitivity to the parent *rad21-K1* (Figure 7D), suggesting that Rad21 also falls in an epistasis group with the Mcm4 C-terminus.

We tested several other mutations affecting proteins associated with the core replisome. Both temperature-sensitive mutations *cdc20-M10* and *pol1-1*, encoding the leading strand DNA polymerase ε (D’Urso and Nurse 1997) and polymerase α (D’Urso *et al.* 1995), respectively, were viable in combination with *mcm4-c106*, while a temperature-sensitive allele *cdc6-23* affecting the lagging strand DNA polymerase δ (Iino and Yamamoto 1997) is lethal (data not shown).

### Mcm4-c106 interactions with repair pathways

Finally, we examined genetic interactions with other mutants in the MMS response pathway. The post replication repair (PRR) pathway includes both error free and error prone branches that facilitate the bypass of base lesions (reviewed in Huang and D’Andrea 2006; Ulrich and Walden 2010). These are regulated by levels of ubiquitylation on PCNA (Frampton 2006). We examined double mutants of *mcm4-c106* with repair mutants *mms2*∆, *ubc13*∆, *pcn1-K164R*, and *rad8*∆. The double mutants were significantly more MMS sensitive than either single mutant, consistent with the mutants working in different pathways. None of these mutants showed a growth defect in the absence of MMS (Figure S5A). Double mutant error prone polymerases *rev3*∆, *rev1*∆, *polκ*∆, or *eso1*-∆ *eta* (Figure S5B) were all more sensitive than the single TLS mutant parents. Interestingly, while the double mutants with *polk* or *rev1*∆ were more sensitive than *mcm4-c106*, double mutants with *eso1*-∆ *eta* or *rev3*∆ were slightly less sensitive. The basis for this difference is unknown.

### Effects of mcm4-c106 in recombination defective mutants

Replication fork stability and fork restart depend on proteins associated with recombination (reviewed in Lambert and Carr 2013; Neelsen and Lopes 2015). We constructed double mutants between *mcm4-c106* and mutations that disrupt recombinational repair of damaged forks including: *mus81*∆ (endonuclease; Boddy *et al.* 2001), *rad50*∆ (MRN complex; Bressan *et al.* 1999), *rad51*∆ (homologous recombination regulator; Muris *et al.* 1993), *rqh1*∆ (RecQ helicase; Stewart *et al.* 1997), and *srs2*∆ (helicase; Wang *et al.* 2001; Maftahi *et al.* 2002). We find that *rad50*∆ *mcm4-c106* is synthetic lethal, indicating that the constitutive damage of *mcm4-c106* requires an active MRN complex for survival. The *mus81*∆ double mutant had an extreme growth defect even at 25°, with slow growth and elongated cell morphology, suggesting the formation of structures that require Mus81 for successful resolution. In contrast, *rad51*∆, *rqh1*∆, or *srs2*∆ double mutants showed no growth defects at permissive temperature, but were sensitive to MMS at a level similar or greater than the most sensitive parent (Figure S6, A and B). These data suggest that these proteins function in a pathway separate from the C-terminus of Mcm4.

## Discussion

Fission yeast Mcm4 is an essential subunit of the MCM helicase, which is a critical component in the response to replication stress. Previous studies have shown that the Mcm4 C-terminal domain (CTD) is important for the efficient recovery of HU-stalled replication forks, and a C-terminal truncation *mcm4-c84* causes excessive formation of ssDNA when replication is inhibited by hydroxyurea (Nitani *et al.* 2008). That study also identified a larger CTD truncation mutant, *mcm4-c106*, as HU-sensitive and temperature-sensitive, but did not characterize it further.

Our initial examination of the temperature-sensitive phenotype of *mcm4-c106* shows three distinct phenotypes that we characterized. First, their temperature-sensitive phenotype has important differences from those in other alleles of *mcm4^+^*. Second, they are MMS-sensitive. Finally, they have distinct replication defects at permissive temperature, including a novel spectrum of genetic interactions

Similar to the original *mcm4-M68* strain (Bailis *et al.* 2008; Sabatinos *et al.* 2015), we observe that *mcm4-c106* cells accumulate a 2C DNA content at restrictive temperature, indicating substantial bulk DNA synthesis (Figure 3B). Cells elongate and do not divide, demonstrating successful activation of the checkpoint, confirmed by a phosphorylation-induced shift of the Chk1 kinase (Figure 4B). The cells also show increased RPA and Rad52 foci during normal growth, temperature shift, and following release, with small punctate morphology similar to that observed in *mcm4-M68* (Figure 5A; Sabatinos *et al.* 2015). Strikingly, however, *mcm4-c106* chromosomes enter a pulsed-field gel normally, both at restrictive temperature and following release, without the chromosome breaks and or structural intermediates that impair chromosome migration in *mcm4-M68*. We previously suggested that the breaks in *mcm4-M68* reflect distinct structures targeted by the Mus81 nuclease; (Sabatinos *et al.* 2015) the intact chromosomes of *mcm4-c106* suggest that whatever structures are formed at restrictive temperature are not the same. The temperature-sensitive phenotype may reflect either Mcm4 protein unfolding (although the protein remains detectable) or some intrinsically temperature-sensitive activity involved in replisome coupling that renders the C-terminus essential at high temperatures. In any case, some fractions of the cells are competent to restart the cell cycle, indicating that the damage they suffer is not irreversible and that the MCM complex remains largely intact and located correctly in the nucleus, which is not seen for *mcm4-M68* (Pasion and Forsburg 1999).

In contrast to the original *mcm4-M68* allele, the *mcm4-c106* cells have clear deficiencies even under permissive conditions. Efficiency of plasmid transformation is a metric for replication efficiency (Clyne and Kelly 1997; Tye 1999), and we observe a substantial reduction in plasmid transformation efficiency and colony size, consistent with a defect in replication in *mcm4-c106* but not in *mcm4-M68* at 25°. In addition, the *mcm4-c106* cells are synthetically lethal with *rad3*∆ or *chk1*∆ checkpoint kinase mutants, and have an increased level of RPA and Rad52 foci at permissive temperature, which suggests that the cells suffer some form of constitutive DNA damage present even at 25°. This is not observed in *mcm4-M68*. We also found synthetic lethality between *mcm4-c106* MRN component *rad50*∆, and severe synthetic sickness with *mus81*∆, which implicates fork processing and restart in the recovery from innate stress. The absence of a synthetic phenotype associated with *rad51*∆ suggests that typical homologous recombination mechanisms are not required.

Interestingly, we found that *mcm4-c106* is also sensitive to alkylation damage caused by MMS treatment at the permissive temperature, unlike other *mcm4* alleles (Figure 1B). Previously, the only MMS-sensitive MCM identified was an allele of *mcm6* (*mcm6-S1*) that is defective in pre-RC assembly (Maki *et al.* 2011). Because *mcm4-c106* cells showed increased sensitivity to MMS in combination with mutations that directly affect downstream repair, including homologous recombination, error free, and error prone postreplication repair pathways, we propose that its defect is not in repair but in fork stability, or restart by template switching.

MMS sensitivity has been observed in mutants affecting a subset of additional replisome components including the fork protection complex (*swi1*∆, *swi3*∆, and *mrc1*∆), scaffolding protein *mcl1*, and the DDK kinase subunits *hsk1-1312* or *dfp1-r35* (Fung *et al.* 2002; Williams and McIntosh 2002; Sommariva *et al.* 2005; Dolan *et al.* 2010). MMS treatment in fission yeast results in slowing of the replication fork (Chahwan *et al.* 2003; Kumar and Huberman 2004; Willis and Rhind 2009). Although there have been reports that MMS generates DNA double strand breaks (Wyatt and Pittman 2006), this breakage is likely be an artifact of the procedure used to extract DNA (Lundin *et al.* 2005), so the PFGE results are likely uninformative. Rather, a major form of recovery is fork arrest, template switching, and repriming (reviewed in Branzei and Foiani 2010), which leads to accumulation of single-stranded DNA and increased recombination intermediates (Willis and Rhind 2009; Koulintchenko *et al.* 2012). Formation of these MMS recombination structures is disrupted in *swi1*∆ and *swi3*∆, and also in *rad2*∆ mutants lacking the FEN1 flap endonuclease (Noguchi *et al.* 2004; Koulintchenko *et al.* 2012). In budding yeast, DNA polymerase α and the Mcl1 ortholog Ctf4 are required for template switching, and evidence indicates that this requirement is not limited to lagging strand lesions (Fumasoni *et al.* 2015). These observations suggest that the scaffolding proteins that link CMG and polymerases are key players to allow successful template switching. The sensitivity of *Mcm4-c106* is unlikely to reflect disruptions in the scaffold itself, as models of CMG suggest that Mcm4 lies on the ring face opposite to Cdc45, GINS, Ctf4, and polymerases (O’Donnell and Li 2016).

We found that *rif1*∆ rescues the MMS sensitivity of *mcm4-c106*, but not its temperature sensitivity. *rif1*∆ also rescues the MMS sensitivity of *swi1*∆ or *swi3*∆. Rif1 in budding yeast has a role in replication timing, via the recruitment of Glc7 phosphatase (Hayano *et al.* 2012; Davé *et al.* 2014; Mattarocci *et al.* 2014; Hiraga *et al.* 2014; Peace *et al.* 2014). This antagonizes the DDK-mediated phosphorylation of Mcm4 that activates replication. Intriguingly, Rif1 is also proposed to modify the response to ssDNA that activates the checkpoint (Xu *et al.* 2010), as well as contributing to resection in break repair (Martina *et al.* 2014). A new study also links Rif1 to resolution of chromosome entanglements (Zaaijer *et al.* 2016). In fission yeast, *rif1*∆ rescues temperature sensitivity of *hsk1-89* (Hayano *et al.* 2012; Davé *et al.* 2014) and *hsk1-1312* (J. P. Yuan and S. L. Forsburg, unpublished data). However, Rif1 is not essential for cellular responses to replication stress (Hayano *et al.* 2012; Peace *et al.* 2014).

There are several models by which this rescue could occur. One way would be by activating otherwise dormant origins, as *rif1*∆ is proposed to deregulate origin timing by antagonizing DDK (Hayano *et al.* 2012). Origin activation has been proposed to rescue the MMS sensitivity of *mcm6-S1*, another MCM subunit (Maki *et al.* 2011). However, unlike *mcm6-S1*, the MMS sensitivity of *mcm4-c106* was not notably rescued by deletion of the S phase cyclin *cig2*, suggesting that additional pre-RC formation is not a mechanism for rescue. Alternatively, rescue may be linked to Rif1 antagonism of the Hsk1/DDK kinase, which is localized to the chromatin in MMS and potentially recruited by the FPC (Sommariva *et al.* 2005; Matsumoto *et al.* 2005; Dolan *et al.* 2010). Hsk1 is known to phosphorylate Mrc1 (associated with FPC) and the checkpoint clamp Rad9 during replication stress (Matsumoto *et al.* 2010; Furuya *et al.* 2010). In the absence of FPC, therefore, loss of localized Hsk1 activity may be balanced by the absence of the Rif1 antagonist.

The similarity of rescue of the MMS phenotype of *mcm4-c106* and *swi1*∆ or *swi3*∆ led us to investigate whether they are in a common epistasis group. Contrary to that model, we observed synthetic lethality between *mcm4-c106* and FPC mutants *swi1*∆, *swi3*∆, and *mrc1*∆, as well as DDK mutants *hsk1-1312* and *dfp1-r35*, indicating that an intact fork-protection complex including DDK must be present in the absence of the Mcm4 C-terminus. A similar synthetic lethal phenotype with the FPC was not observed with *mcm4-c84*, which harbors a shorter truncation of the C-terminus (Nitani *et al.* 2008), so this is unlikely to be related to the HU-ssDNA phenotype observed in that study.

Because we see no phenotype in double mutants with the replication checkpoint kinase *cds1*∆, we conclude that this synthetic lethality is not related to the S phase checkpoint activation by FPC (Noguchi *et al.* 2003), but rather to the FPC’s structural role linking the helicase to the polymerase (Noguchi *et al.* 2004; Lou *et al.* 2008; reviewed in Aze *et al.* 2013).

Mcl1 (ScCtf4) links the helicase to the lagging strand through the interaction with polymerase α and direct interaction with the GINS subcomplex (Gambus *et al.* 2009; Simon *et al.* 2014). At least one arm of the helicase coupling system is required for viability, because *mcl1-1 swi1*∆ double mutants are synthetically lethal (J. P. Yuan and S. L. Forsburg, unpublished results). Therefore, one possibility is that mutation of *mcm4-c106* may lead to defects in coupling to the lagging strand side of the replisome, making it dependent upon the FPC and the leading strand coupling for viability even at permissive temperature.

FPC mutants are also synthetically lethal, with mutations affecting Ctf18 and Ctf8 forming an alternative RFC clamp loader complex (Ansbach *et al.* 2008). Long linked to sister chromatid cohesion (Hanna *et al.* 2001; Mayer *et al.* 2001), Ctf18 has more recently been associated with DNA damage response and replication checkpoint activation (Crabbé *et al.* 2010; Kubota *et al.* 2011). We observed modestly reduced permissive temperature in *ctf18*∆ *mcm4-c106*, but no defect at permissive temperature and little change in MMS sensitivity, which suggests that they are in a common epistasis group at this temperature. Double mutants between *mcm4-c106* and the *mcl1-1* temperature-sensitive allele were viable, although significantly sicker than either parent. Our genetic analysis offers further tantalizing evidence that this phenotype may be linked to cohesion. FPC, Mcl1, polε, DDK, and Ctf18 are all associated with sister chromatid cohesion in *S. pombe* and other systems (Hanna *et al.* 2001; Williams and McIntosh 2002; Bailis *et al.* 2003; Edwards *et al.* 2003; Xu *et al.* 2007; Ansbach *et al.* 2008; Rapp *et al.* 2010). We observed no evidence for chromosome segregation errors in *mcm4-c106* that would indicate a sister chromatid cohesion defect. A temperature-sensitive mutation affecting the cohesin subunit *rad21-K1* is synthetic lethal with either *swi1*∆ (Ansbach *et al.* 2008; Dolan *et al.* 2010) or *hsk1-1312* (Snaith *et al.* 2000). In contrast to the substantial genetic interactions in other double mutants, we did not observe growth defects in *rad21-K1 mcm4-c106*. Additionally, the MMS sensitivity of *rad21 mcm4-c106* is similar to that of the *rad21-K1* parent. We propose that the C-terminus of Mcm4 may facilitate the recruitment of cohesin to promote a distinct fork maintenance or restart function.

As its name implies, the *S. pombe* cohesin *rad21* gene was first identified by its repair defect (Birkenbihl and Subramani 1992). Mutations in *rad21* are MMS-sensitive, and lie in an epistatic pathway with *rad50* (Hartsuiker *et al.* 2001). More recently, studies suggest that cohesin may influence replication origin activity by affecting 3-D genome organization (Guillou *et al.* 2010; Yun *et al.* 2016). Cohesin has been shown to be required for efficient template switching in budding yeast in a pathway that includes Ctf4 (*Sp* Mcl1) (Fumasoni *et al.* 2015). Particularly intriguing, Mcm4 has also been identified as a binding partner of mammalian Rad21 in two separate proteomics studies (Guillou *et al.* 2010; Panigrahi *et al.* 2012). We suggest that the extended C-terminus of Mcm4 collaborates with cohesin to promote fork stability during replication stress.

Together, these results indicate that *mcm4-c106* has a novel replication defect, likely to do with replisome uncoupling, that is distinct from that in other *mcm4* conditional alleles. Along with our previous study (Sabatinos *et al.* 2015), this suggests that physiological inspection of conditional mutant phenotypes is likely to identify new domains and interactions that assemble and maintain the replicative helicase.

## 

## Supplementary Material

Supplemental Material
