# Supplementary material for: Characterization of a Novel MMS-Sensitive Allele of Schizosaccharomyces pombe mcm4+
Source: G3 (Bethesda). 2016 Jul 29;6(10):3049–63. doi: 10.1534/g3.116.033571 (PMC5068930; doi:10.1534/g3.116.033571)
Supplement: Supplemental Material [file supp_6_10_3049__index.html]

Characterization of a Novel MMS-Sensitive Allele ofSchizosaccharomyces pombe mcm4+ — Supplemental Material 

# Characterization of a Novel MMS-Sensitive Allele of *Schizosaccharomyces pombe mcm4+*

## Supplemental Material for Ranatunga, *et al*, 2016

**Files in this Data Supplement:**

- Figure S6 - *mcm4-c106* interactions with genes involved in recombination and other pathways. (.pdf, 546 KB)
- Figure S1 - Viability of mcm4 mutants treated with HU and CPT. (.jpg, 298 KB)
- Figure S2 - Chromosomes fail to enter gel following MMS treatment and release. (.jpg, 122 KB)
- Figure S3 - *mcm4c-106* chromosome segregation during mitosis at 36° using LacI LacO system. (.jpg, 343 KB)
- Figure S4 - Effects of *Cig2Δ* on *mcm4-c106* MM S sensitivity. (.jpg, 171 KB)
- Figure S5 - *mcm4-c106* interactions with genes involved in the error free and error prone repair pathways. (.jpg, 297 KB)
