# Supplementary figures and images for: Characterization of a Novel MMS-Sensitive Allele of Schizosaccharomyces pombe mcm4+
Source: G3 (Bethesda). 2016 Jul 29;6(10):3049–63. doi: 10.1534/g3.116.033571 (PMC5068930; doi:10.1534/g3.116.033571)

Figure S6A

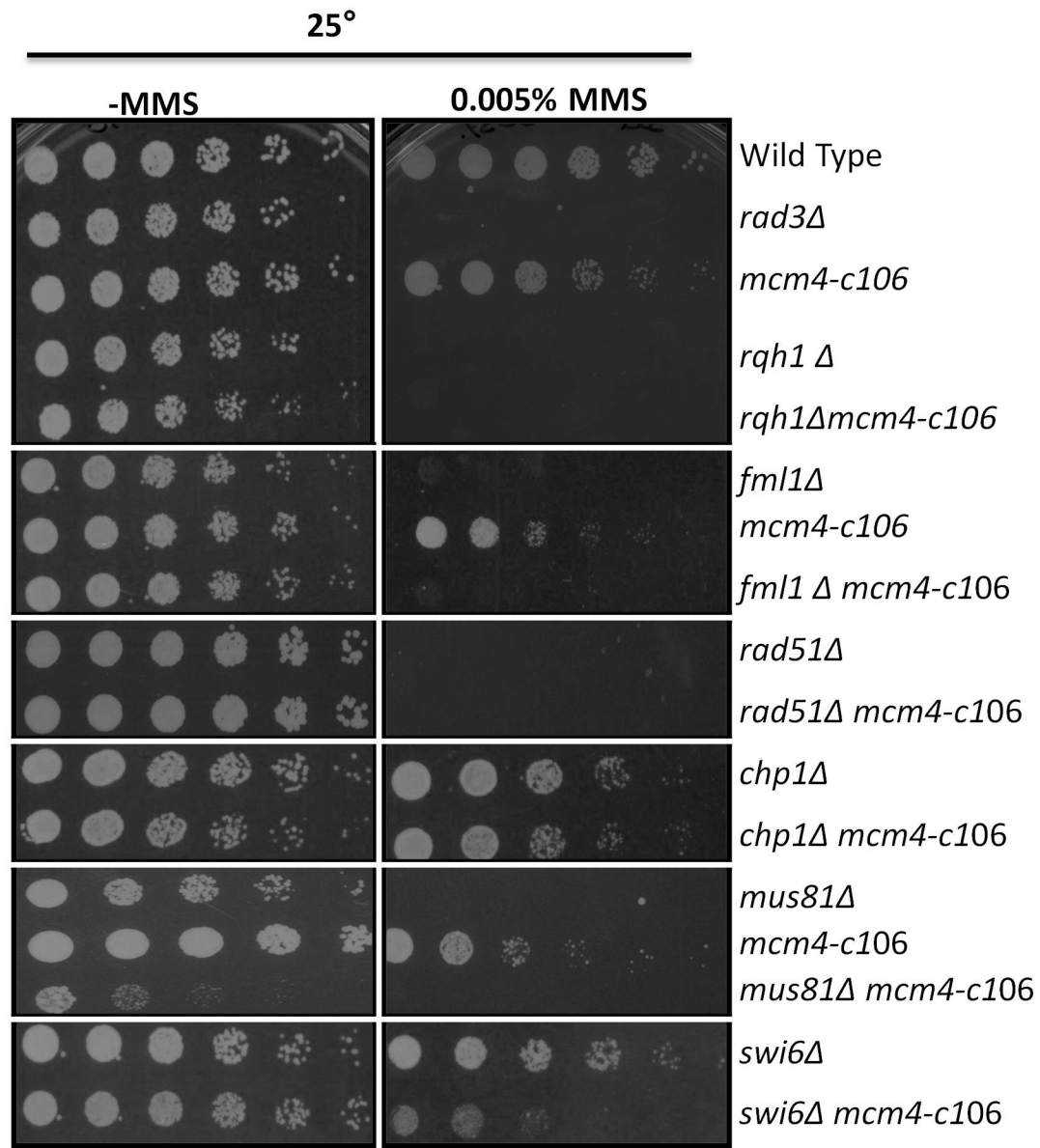

Figure S6B

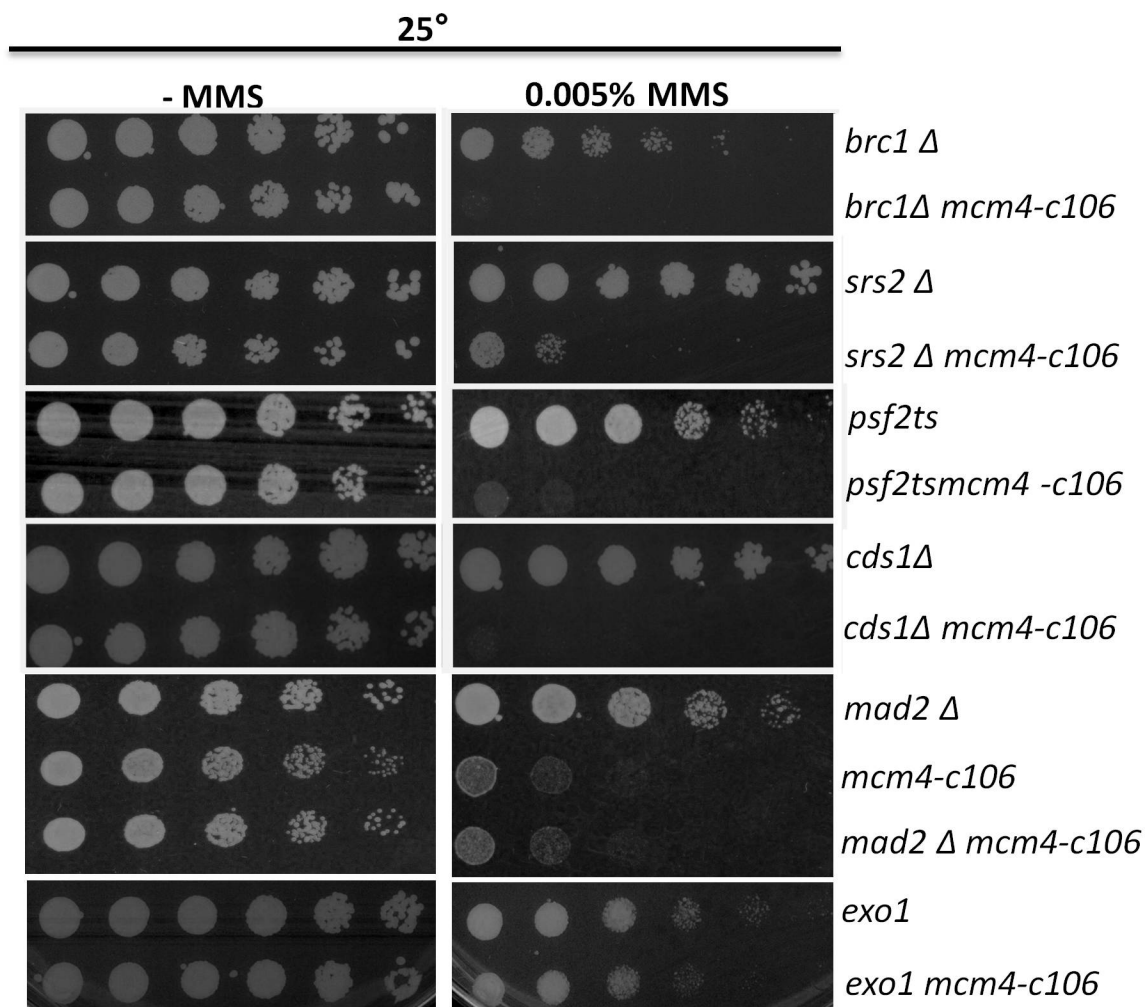

Supplement: Supplemental Material [file supp_g3.116.033571_FigureS6.pdf]

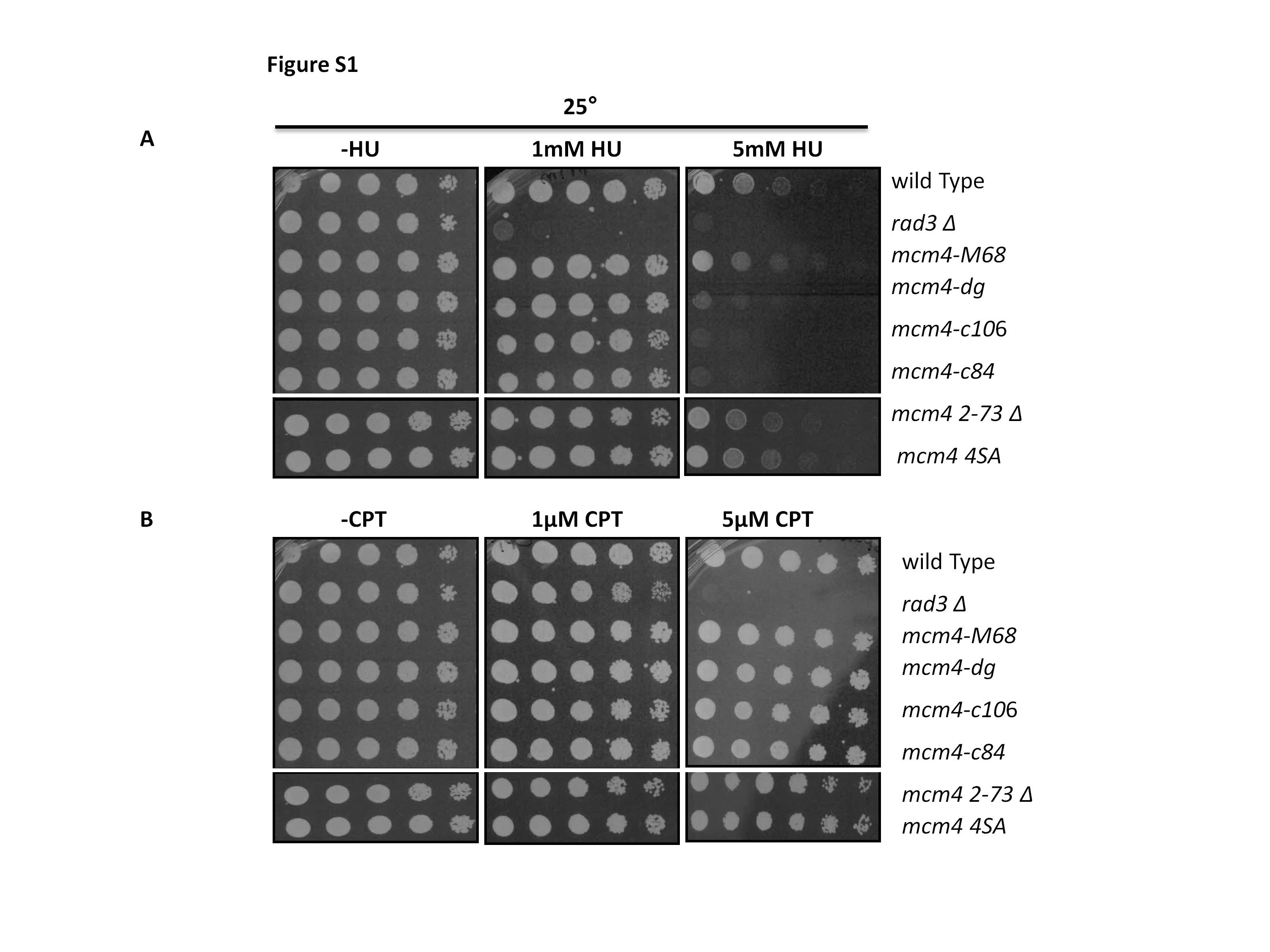

Supplement: Supplemental Material [file supp_g3.116.033571_FigureS1.jpg]

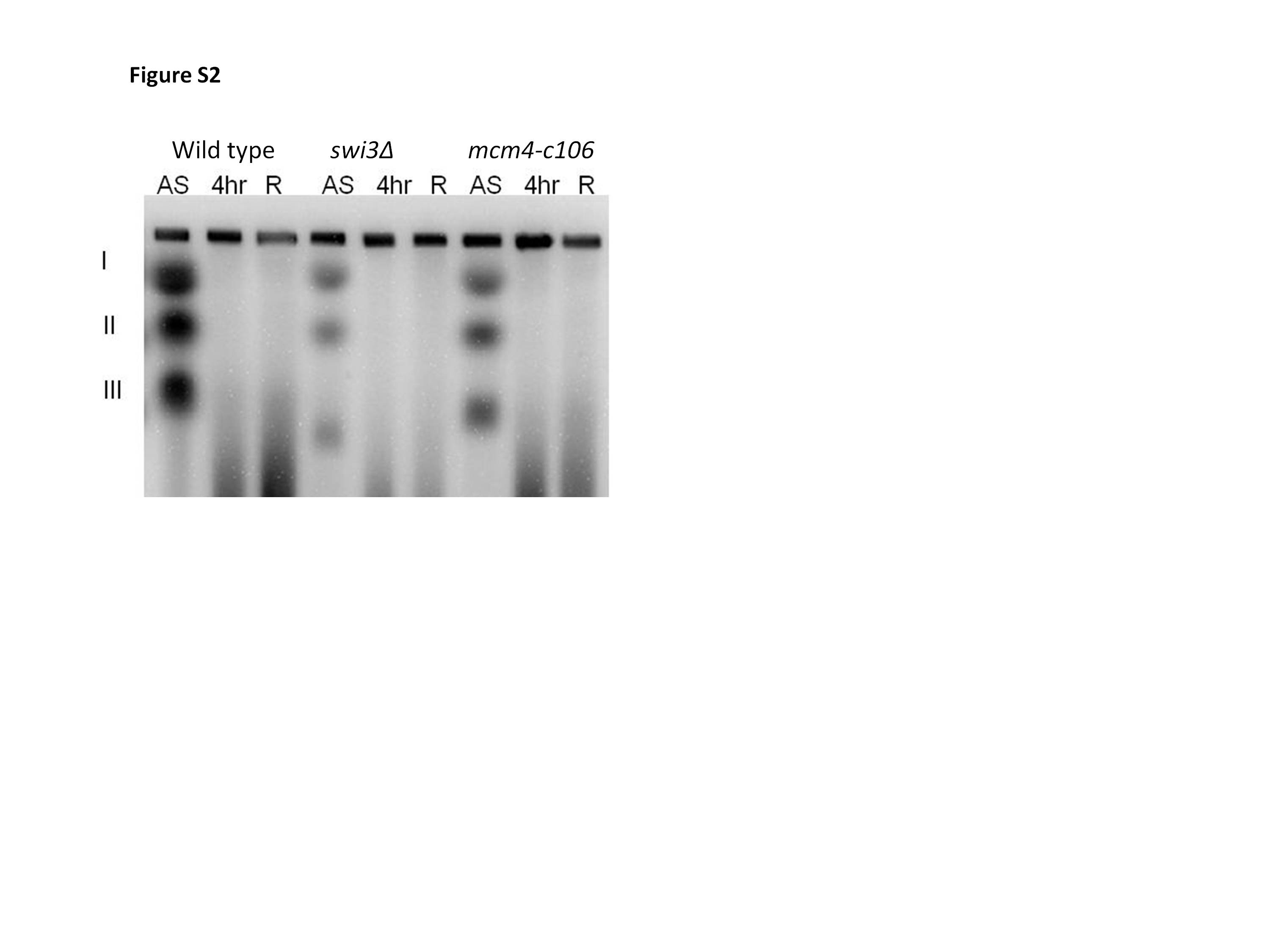

Supplement: Supplemental Material [file supp_g3.116.033571_FigureS2.jpg]

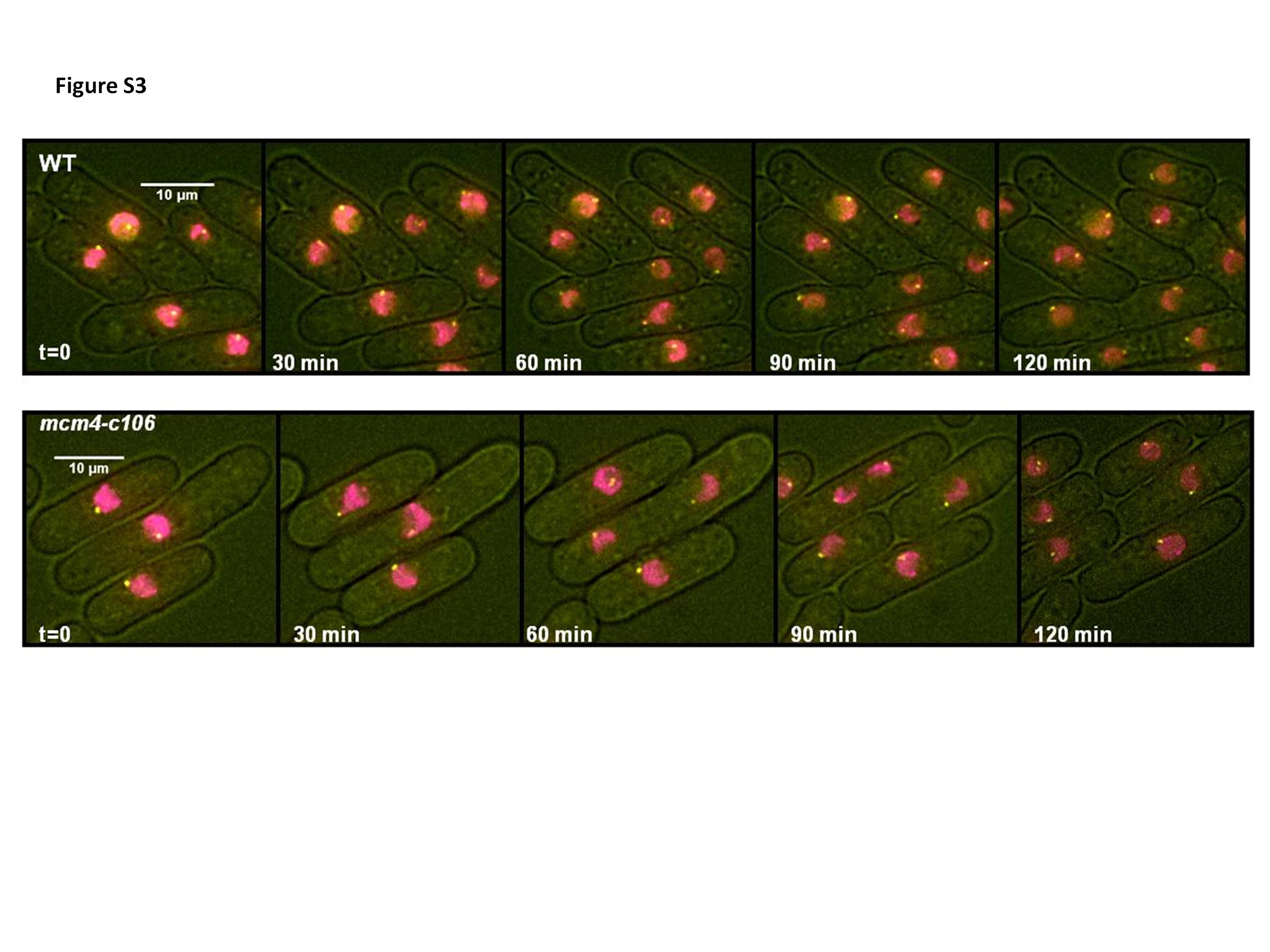

Supplement: Supplemental Material [file supp_g3.116.033571_FigureS3.jpg]

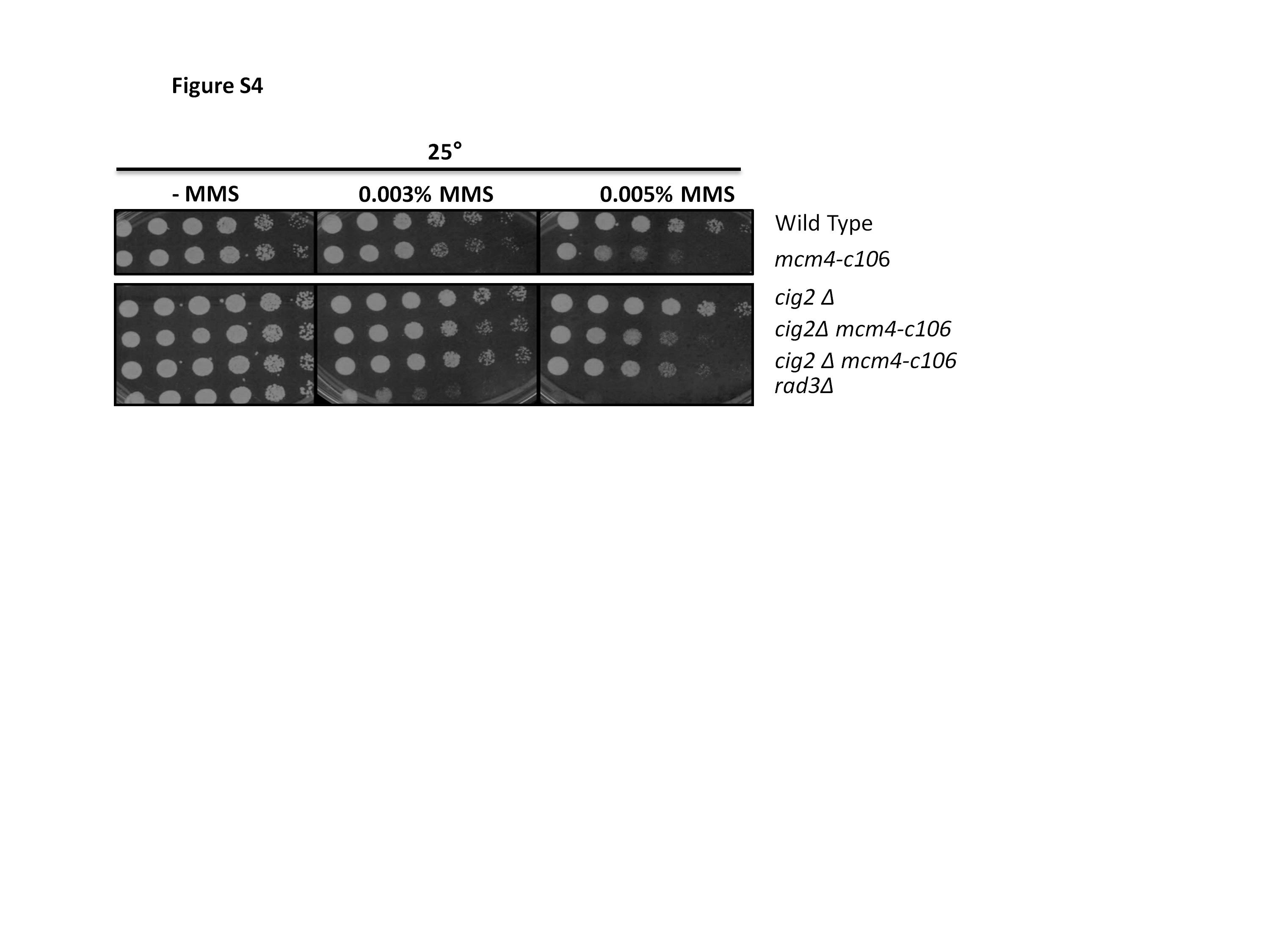

Supplement: Supplemental Material [file supp_g3.116.033571_FigureS4.jpg]

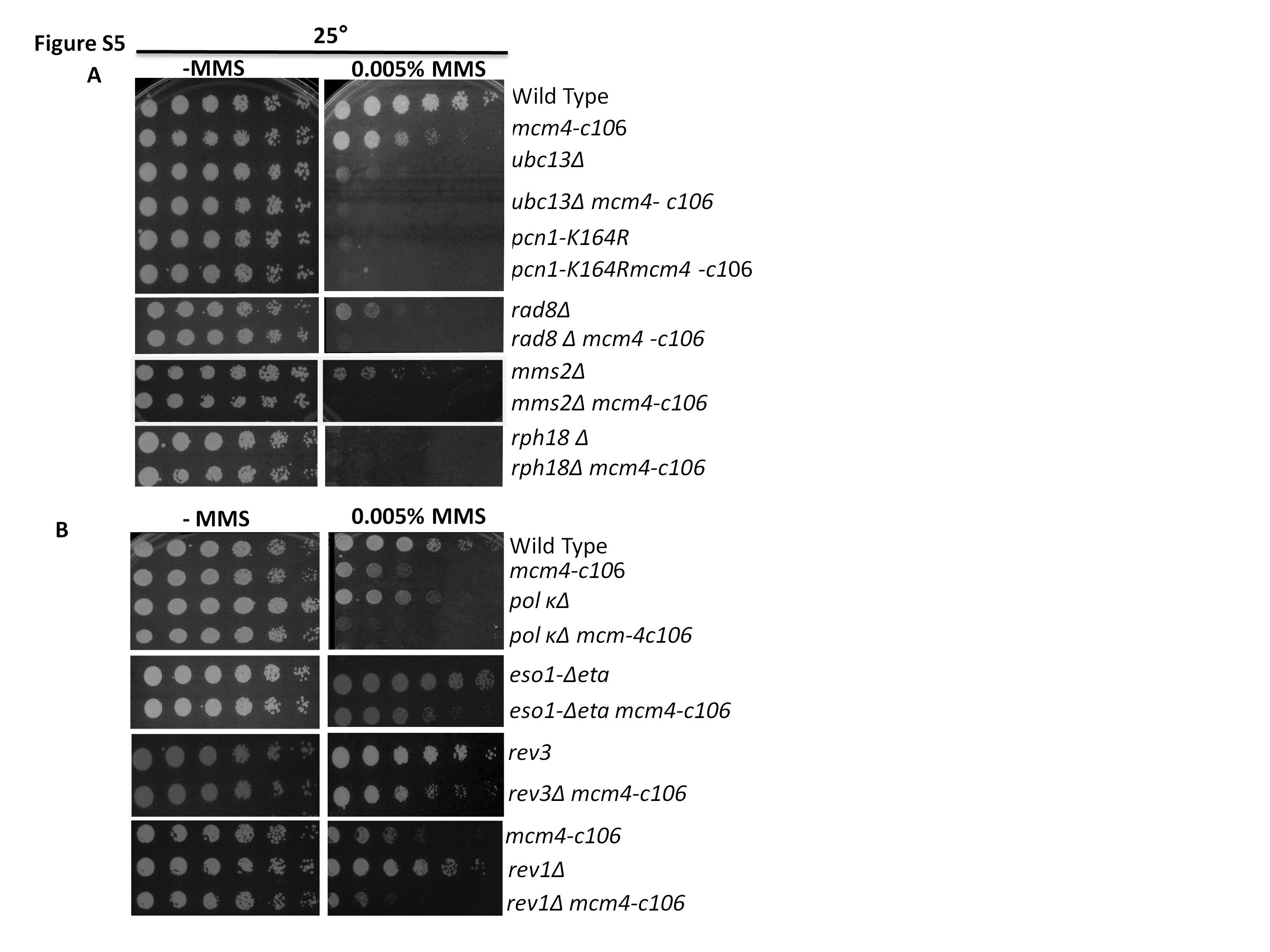

Supplement: Supplemental Material [file supp_g3.116.033571_FigureS5.jpg]
